# The Isolate *Caproiciproducens* sp. 7D4C2 Produces *n*-Caproate at Mildly Acidic Conditions From Hexoses: Genome and rBOX Comparison With Related Strains and Chain-Elongating Bacteria

**DOI:** 10.3389/fmicb.2020.594524

**Published:** 2021-01-14

**Authors:** Sofia Esquivel-Elizondo, Caner Bağcı, Monika Temovska, Byoung Seung Jeon, Irina Bessarab, Rohan B. H. Williams, Daniel H. Huson, Largus T. Angenent

**Affiliations:** ^1^AG Angenent, Max Planck Institute for Developmental Biology, Max Planck Society (MPG), Tübingen, Germany; ^2^Algorithms in Bioinformatics, Department of Computer Science, University of Tübingen, Tübingen, Germany; ^3^International Max Planck Research School “From Molecules to Organisms”, Max Planck Institute for Developmental Biology, University of Tübingen, Tübingen, Germany; ^4^Environmental Biotechnology Group, Center for Applied Geosciences, University of Tübingen, Tübingen, Germany; ^5^Integrative Analysis Unit, Singapore Centre for Environmental Life Sciences Engineering, National University of Singapore, Singapore, Singapore

**Keywords:** chain elongation, *n*-caproate, lactate, reverse β-oxidation, rBOX genes, *Caproiciproducens*, chain-elongating bacteria, thiolase

## Abstract

Bulk production of medium-chain carboxylates (MCCs) with 6–12 carbon atoms is of great interest to biotechnology. Open cultures (e.g., reactor microbiomes) have been utilized to generate MCCs in bioreactors. When in-line MCC extraction and prevention of product inhibition is required, the bioreactors have been operated at mildly acidic pH (5.0–5.5). However, model chain-elongating bacteria grow optimally at neutral pH values. Here, we isolated a chain-elongating bacterium (strain 7D4C2) that grows at mildly acidic pH. We studied its metabolism and compared its whole genome and the reverse β-oxidation (rBOX) genes to other bacteria. Strain 7D4C2 produces lactate, acetate, *n*-butyrate, *n*-caproate, biomass, and H_2_/CO_2_ from hexoses. With only fructose as substrate (pH 5.5), the maximum *n*-caproate specificity (i.e., products *per* other carboxylates produced) was 60.9 ± 1.5%. However, this was considerably higher at 83.1 ± 0.44% when both fructose and *n*-butyrate (electron acceptor) were combined as a substrate. A comparison of 7D4C2 cultures with fructose and *n*-butyrate with an increasing pH value from 4.5 to 9.0 showed a decreasing *n*-caproate specificity from ∼92% at mildly acidic pH (pH 4.5-5.0) to ∼24% at alkaline pH (pH 9.0). Moreover, when carboxylates were extracted from the broth (undissociated *n*-caproic acid was ∼0.3 mM), the *n*-caproate selectivity (i.e., product *per* substrate fed) was 42.6 ± 19.0% higher compared to 7D4C2 cultures without extraction. Based on the 16S rRNA gene sequence, strain 7D4C2 is most closely related to the isolates *Caproicibacter fermentans* (99.5%) and *Caproiciproducens galactitolivorans* (94.7%), which are chain-elongating bacteria that are also capable of lactate production. Whole-genome analyses indicate that strain 7D4C2, *C. fermentans*, and *C. galactitolivorans* belong to the same genus of *Caproiciproducens*. Their rBOX genes are conserved and located next to each other, forming a gene cluster, which is different than for other chain-elongating bacteria such as *Megasphaera* spp. In conclusion, *Caproiciproducens* spp., comprising strain 7D4C2, *C. fermentans*, *C. galactitolivorans*, and several unclassified strains, are chain-elongating bacteria that encode a highly conserved rBOX gene cluster. *Caproiciproducens* sp. 7D4C2 (DSM 110548) was studied here to understand *n*-caproate production better at mildly acidic pH within microbiomes and has the additional potential as a pure-culture production strain to convert sugars into *n*-caproate.

## Introduction

Medium-chain carboxylates (MCCs, 6–12 carbon atoms) are precursors to liquid fuels (Levy et al., 1981). Production of MCCs is, therefore, of great interest to biotechnology as a production platform for large volumes, especially since the substrate can be organic wastes or wastewater as part of the circular economy. MCCs are much easier to separate from the culture broth compared to short-chain carboxylates (SCCs, 2–5 carbon atoms) due to their hydrophobic carbon chains (Levy et al., 1981; Xu et al., 2015; Angenent et al., 2016). Besides their use for fuel production, MCCs are also feedstocks in the chemical, pharmaceutical, food, and agricultural industries for the manufacture of a wide variety of products (Levy et al., 1981; Kenealy et al., 1995; Desbois, 2012; Harvey and Meylemans, 2014). Moreover, MCCs are used for food preservation and sanitation due to their antimicrobial properties at low pH (Harroff et al., 2017).

Carboxylates exist in an undissociated (carboxylic acid) and dissociated form (conjugate base, or carboxylate, plus a proton), depending on the pH of the bioreactor broth. At mildly acidic pH, specifically below the pKa (∼4.9), the carboxylic acid is in the undissociated form. At pH values higher than the pKa, the acid dissociates and releases one proton, forming the conjugate base. The undissociated form of a carboxylate (i.e., the carboxylic acid) is hydrophobic, which is essential for separation, but it is also lipophilic and crosses the microbial cell wall, creating antimicrobial properties. Inside the cell, where the pH is higher than in the bioreactor broth, the acid dissociates. As the conjugate base is lipophobic, it accumulates inside the cell, resulting in microbial inhibition (Russell, 1992). Based on this, *n*-caproate, which is a 6-carbon MCC (here referred to as the total of dissociated and undissociated forms), is toxic to microbes at pH values near its pKa (Agler et al., 2012a; Ge et al., 2015).

**FIGURE 1:**
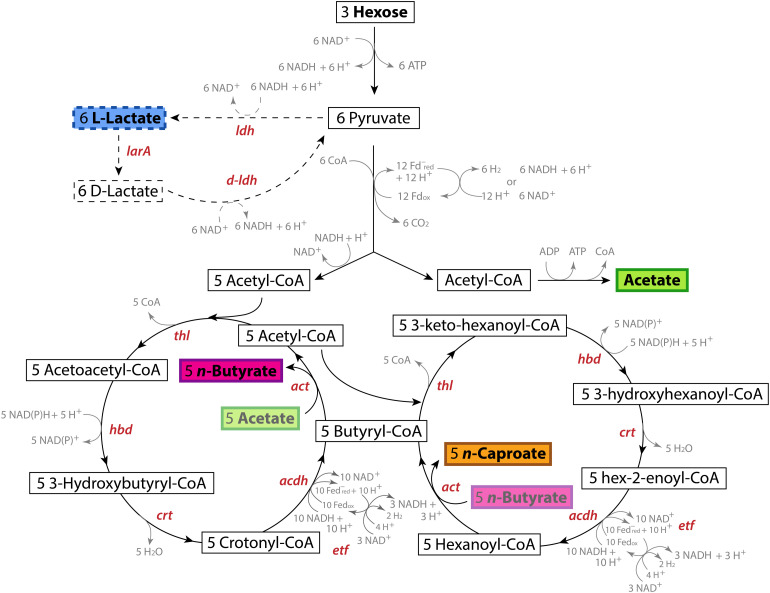
Pathways and genes involved in the conversion of hexoses into lactate and the conversion of these substrates into *n*-caproate *via* the reverse β-oxidation (rBOX) pathway. The first cycle of the rBOX pathway involves the conversion of the acetate produced by one acetyl-CoA molecule into *n*-butyrate. The second cycle involves the conversion of this *n*-butyrate into *n*-caproate *via* the butyryl-CoA produced in the first cycle and an acetyl-CoA molecule. The genes that code for the enzymes catalyzing the production of lactate and its conversion into pyruvate and each reaction of the rBOX pathway are shown for each reaction. rBOX genes: *thl*, thiolase (acetyl-CoA C-acetyltransferase); *hbd*, 3-hydroxybutyryl-CoA dehydrogenase; *crt*, crotonase (3-hydroxybutyryl-CoA dehydratase); *acdh*, Acyl-CoA dehydrogenase; *etf*, Electron transport flavoprotein; *act*, acetate-CoA transferase. Lactate production gene: *ldh*, *L-ldh*, L-lactate dehydrogenase. Lactate consumption genes: *larA*, lactate racemase; *D-ldh*: D-lactate dehydrogenase.

Chain-elongating bacteria produce MCCs *via* the rBOX pathway. In this strictly anaerobic process, electron donors, such as fructose, sucrose, lactate, or ethanol, are oxidized into several acetyl-CoA molecules (2 carbons each). A certain fraction of these molecules is converted to produce acetate and energy. The other fraction of the acetyl-CoA molecules is used to elongate acetate or other SCCs (electron acceptors) in a cyclic process where two carbons are added at a time (Figure 1). In this manner, acetate (2 carbons) is first elongated to *n*-butyrate (4 carbons) and then to *n*-caproate (6 carbons). In some cases, *n*-caprylate (8 carbons) is produced (Spirito et al., 2014; Kucek et al., 2016a,b). When propionate is the electron acceptor, *n*-valerate (5 carbons) and *n*-heptanoate (7 carbons) are produced (Jeon et al., 2016). However, electron donors can also be used solely to produce MCCs (Jeon et al., 2010). The key enzymes involved in the rBOX pathway are thiolase (Thl; also named acetyl-CoA C-acetyltransferase), HBD, crotonase (Crt; also named 3-hydroxybutyryl-CoA dehydratase), ACDH, ETF, and acetate-CoA transferase (ACT) (Figure 1).

Open cultures (e.g., reactor microbiomes) have been used to generate MCCs at high rates from various synthetic feeds and industrial and agricultural wastewaters, which are rich in carbon and electron equivalents such as sugar-rich and lactate-rich effluents (Kucek et al., 2016a; Duber et al., 2018; Xu et al., 2018; Contreras-Dávila et al., 2020). These bioreactors are operated: (1) at neutral pH to circumvent the accumulation of the undissociated form of the carboxylates, or (2) at mildly acidic pH (5.0–5.5) with in-line MCC extraction to recover the carboxylate product and to prevent product inhibition. The operation of bioreactors at mildly acidic pH values has the advantage of facilitating the extraction of MCCs from the culture broth because, at these pH values, MCCs have a low maximum solubility (Xu et al., 2015). Also, the low pH in open-culture bioreactors inhibits acetoclastic methanogenesis, which would be the main, but unwanted, electron shunting mechanism in reactors operated at neutral pH (Ge et al., 2015).

To increase the likelihood that MCC production in bioreactors with in-line extraction becomes an economic proposition as a biotechnology production platform, it is essential to study chain-elongating bacteria that thrive under mildly acidic conditions. A few chain-elongating bacteria have been isolated. *Clostridium kluyveri* is the most studied chain-elongating bacterium and known to utilize ethanol as the primary electron donor (Angenent et al., 2016). Other well-studied chain-elongating bacteria use carbohydrates (e.g., *Caproiciproducens galactitolivorans*, *Megasphaera hexanoica*, *Megasphaera elsdenii*, *Megasphaera indica*) or lactate (e.g., *Ruminococcaceae* bacterium CPB6 and *M. elsdenii*) as electron donors (Marounek et al., 1989; Roddick and Britz, 1997; Lanjekar et al., 2014; Jeon et al., 2016; Zhu et al., 2017). Recently, *Caproicibacter fermentans*, which is an *n*-caproate producer from carbohydrates, was isolated (Flaiz et al., 2020). While open cultures can effectively perform chain elongation at mildly acidic pH conditions with in-line MCCs extraction, strain CPB6 and *C. fermentans* are the only known chain-elongating bacteria that can satisfactorily produce MCCs at mildly acidic pH levels (Zhu et al., 2017; Flaiz et al., 2020).

Whole-genome analyses combined with laboratory experiments are a powerful approach to study chain-elongating bacteria. While whole-genome alignments are necessary to assign taxonomy to novel microbes, the presence and location of genes give insights into their metabolism. The main objective of this work was to isolate and study the metabolism of a chain-elongating bacterium that thrives at mildly acidic pH (>4.5). To consider its potential application in bioreactors that are aimed at MCC production, we identified the environmental conditions that enhanced its *n*-caproate production. We sequenced and assembled its whole genome and compared it to other bacteria to assign taxonomy. We focused our comparisons on its closest isolated relatives *C. fermentans* (99.5% similar based on the 16S rRNA gene sequence) and *C. galactitolivorans* (94.71%), and also on unclassified strains. Moreover, we studied the genes encoding rBOX proteins (rBOX genes) and compared them to those in: (1) close relatives; (2) bacteria with similar rBOX genes; and (3) known-chain-elongating bacteria.

## Materials and Methods

### Isolation of Strain 7D4C2

Rumen fluid (from a young sheep) and thermophilic anaerobic sludge, which was collected at the Western Lake Superior Sanitary District in 2011 (Duluth, MN, United States), were used to inoculate a bioreactor converting pretreated cellulosic hydrolysate into *n*-butyrate (Agler et al., 2012b). Mixed liquor from this bioreactor was used to start a chain-elongation study with ethanol beer (Agler et al., 2012a; Ge et al., 2015). After 5 years of chain elongation with ethanol beer, the mixed liquor was used to inoculate three chain-elongating bioreactors producing *n*-caproate and *n*-caprylate from ethanol and acetate (Spirito et al., unpublished data). We used a cryogenic sample from one of these reactors to isolate bacteria *via* soft agar serial dilutions, as indicated in Supplementary Figure 1. For this, 10 mL of sterile and reduced supplemented basal medium (Supplementary Table 1), containing 0.6% w/v Bacto Agar (Becton Dickinson, Sparks, MD, United States), were dispensed in 15-mL test tubes that were capped with butyl rubber stoppers and screw caps. After 1–2 weeks of incubation at 30°C and a pH of 5.2 ± 0.1, we picked single colonies in an anaerobic glove box (MBraun, Garching, Germany). We cultured the selected colonies in 10 mL of supplemented basal medium with ethanol (Sigma-Aldrich, Steinheim, Germany) and/or fructose (Carl Roth, Karlsruhe, Germany) as substrates in 50-mL serum bottles. After 1–2 weeks of cultivation (when the cultures were turbid), we measured *n*-caproate and H_2_ production and substrate consumption. The purity of cultures that produced *n*-caproate was examined through scanning electron and/or light microscopy and Sanger sequencing. The isolate that showed 100% purity is referred to as strain 7D4C2.

### Cultivation of Strain 7D4C2

We evaluated the chain-elongating metabolism of strain 7D4C2 with different electron acceptors, as well as its *n*-caproate and lactate production at different pH values and temperatures. For these, we grew strain 7D4C2 in 50-mL serum bottles with 10 mL of supplemented basal medium buffered with 93.18 ± 6.85 mM MES (Carl Roth, Karlsruhe, Germany) (Supplementary Table 1). For the electron-acceptor experiment (30°C, pH 5.5 ± 0.02), we used 24.4 ± 1.7 mM fructose (146.4 ± 10.3 mmol C L^–1^) and the following carboxylates at a concentration of 108.2 ± 8.0 mmol C L^–1^: Na-acetate (VWR, Solon, OH, United States), Na-butyrate, propionic acid (Merck, Darmstadt, Germany), *n*-valeric acid (Merck, Darmstadt, Germany) and *n*-caproic acid (Carl Roth, Karlsruhe, Germany). This experiment was performed in triplicate. In the experiments at different pH values and temperatures, the primary substrates were 24.7 ± 0.5 mM fructose (148.2 ± 3.2 mmol C L^–1^) and 18.7 ± 1.0 mM Na-butyrate (112.2 ± 6.3 mmol C L^–1^) (Thermo Fisher, Kandel, Germany). For the pH experiment, we grew strain 7D4C2 at 30°C in a pH range from 4.5 to 10. The initial pH value was adjusted with 2 N sodium hydroxide (Sigma-Aldrich, Steinheim, Germany). For the temperature test, we grew strain 7D4C2 at various temperatures (i.e., 22.5, 27, 30, 37, 42, and 50°C) at the previously determined optimum pH value (i.e., pH of 6.0). These experiments were performed in duplicate.

### Extraction of *n*-Caproate With Mineral Oil and 3% (w/v) TOPO

To assess whether the bacterium could produce more *n*-caproate without the inhibition of the undissociated acid, we continuously extracted the MCC using an extraction solvent. The extraction solvent consisted of 30 g/L of tri-*n*-octylphosphine oxide (TOPO, Acros Organics, Geel, Belgium) in mineral oil (Sigma-Aldrich, Steinheim, Germany) (Kucek et al., 2016a). For this experiment, we grew strain 7D4C2 in 50-mL serum bottles containing 10 mL of supplemented basal medium (314.1 ± 2.1 mmol C L^–1^ fructose, 101.3 ± 3.2 mmol C L^–1^ Na-butyrate, pH 5.2) (Supplementary Table 1). We added 10 mL of UV light- sterilized extraction solvent after 3 days of growth, when the *n*-caproate concentration was increasing, to prevent the initial loss of substrate (i.e., *n*-butyrate) into the extractant. The solvent preferentially extracts hydrophobic molecules, resulting in extraction efficiencies of 83–93% for MCCs and 5–31% for SCCs (Agler et al., 2012b). Because *n*-caproate is more hydrophobic than *n*-butyrate when *n*-caproate is present, it is the main carboxylate extracted. The control 7D4C2 cultures did not include an extraction solvent. Along with the addition of extractant, we added ∼30 mM more fructose into all 7D4C2 cultures to promote *n*-caproate production. We calculated the concentration of undissociated acid using the Henderson–Hasselbalch equation (Harroff et al., 2017). We took liquid samples (0.6 mL) from the culture and solvent phases. We washed the solvent samples five times with an equal amount of 0.3 M sodium borate (Acros Organics, Geel, Belgium) (pH = 9) to back-extract the carboxylic acids. The aqueous phase (i.e., boric acid with the extracted carboxylates) of each wash was analyzed as indicated below. The concentrations from each washing were summed to estimate the carboxylate production/consumption per data point. We tested these experiments in triplicate at 30°C.

### Comparison Among Strain 7D4C2, *C. galactitolivorans*, and [*C.*] *leptum*

*Caproiciproducens galactitolivorans* BS-1 was acquired from the Japan Collection of Microorganisms RIKEN and [*C*.] *leptum* VPI T7-24-1 from the German Collection of Microorganisms and Cell Cultures (DSMZ). The sugar consumption of strain 7D4C2, C. *galactitolivorans*, and *C. leptum* was compared in 50-mL serum bottles incubated at 37°C and a pH of 7.0. Since *C. leptum* did not grow in the supplemented basal medium in which we grew strain 7D4C2 (Supplementary Table 1), nor in the optimized medium for *C. galactitolivorans* (Jeon et al., 2013), the three bacteria were grown in 10 mL of DSMZ medium 107c with glucose as the primary substrate. We tested these experiments in triplicate.

### Analysis of Sugars, Carboxylates, and H_2_

We quantified sugars and carboxylates (the total of the dissociated and undissociated forms) throughout the culturing period *via* high-performance liquid chromatography (HPLC), as described in Klask et al. (2020). For the sample preparation, 0.6 mL of culture were centrifuged at 13,350 rpm for 6 min in a Benchtop centrifuge (5424 Eppendorf, Hamburg, Germany). The supernatant was filtered through a 0.22-μm polyvinylidene fluoride syringe filter (Carl Roth, Karlsruhe, Germany) and stored alongside the biomass pellets at −20°C until analyzed. Only the acetate, *n*-butyrate, and *n*-caproate concentrations from the pH experiment were analyzed with an Agilent 7890B gas chromatograph (Agilent Technologies, Inc., Santa Clara, CA, United States), which was equipped with a capillary column (DB-Fatwax UI 30 m × 0.25 m; Agilent Technologies) and an FID detector with a ramp temperature program (initial temperature of 80°C for 0.5 min, then 20°C per min up to 180°C, and final temperature of 180°C for 1 min). The injection and detector temperatures were 250 and 275°C, respectively. Samples were prepared as for HPLC with the addition of an internal standard (Ethyl-butyric acid) and acidification (to pH 2) with 50% formic acid.

To assess H_2_ production, we collected 250-μL gas samples with a 500-μL syringe (Hamilton, Giarmata, Romania). We injected 200 μL in a gas chromatograph (SRI 570 8610C, SRI Instruments, Las Vegas, NV, United States) with the characteristics described in Ruaud et al. (2020). We used the ideal gas equation to calculate the moles of H_2_ produced per culture volume. For this, we measured the gas pressure in the serum bottles with a digital pressure gauge (Cole Parmer, Vernon Hills, IL, United States). We measured the cell density (OD_600_) with a NanoPhotometer NP80 at 600 nm with a path length of 0.67 mm (Implen, Westlake Village, CA, United States).

### Microscopy and Morphology Characterization

To image the isolate *via* light microscopy, we centrifuged a 0.5-mL sample of culture in the exponential phase at 7,000 rpm for 5 min in a Benchtop centrifuge (5424 Eppendorf, Hamburg, Germany). We washed the pelleted biomass 1–2 times and resuspended it with 50 μL 1× PBS from which we fixed 2 μL on solidified agarose (VWR, Solon, OH, United States) (1% w/v). To image the isolate *via* scanning electron microscopy (SEM), we pelleted 6 mL of culture for 3 min at 7000 rpm (Benchtop centrifuge 5424 Eppendorf, Hamburg, Germany) inside a glove-box (MBraun, Garching, Germany). We washed the pellet five times with 500 μL of 1× PBS. After the last washing step, we resuspended the pellet with 450 μL of 1× PBS and added 50 μL of 25% (v/v) glutaraldehyde for fixation. Samples were incubated at room temperature for 2 h, and then handed over to the SEM center at the Max-Planck Institute for Developmental Biology (Tübingen, Germany) for further processing and imaging, as detailed in Ruaud et al. (2020). For Gram staining, we used the Gram stain for films kit (Sigma-Aldrich, Steinheim, Germany), as described in the manufacturer’s protocol.

### DNA Extraction and 16S rRNA Gene Sequence Phylogenetic Analysis

We extracted DNA from the biomass pellets stored at −20°C using a NucleoSpin® Microbial DNA Kit (Macherey-Nagel, Düren, Deutschland), according to the manufacturer’s protocol. The 16S rRNA gene was amplified from genomic DNA using the universal primers sets 27F/1391R and 27F/1525R. The PCR product was purified with DNA Clean Concentrator-5 (Zymo Research, Irvine, CA, United States). Universal primers 27F, 342F, 515F, 926F, and 926R and the designed primer 1492-capro-R (CTACCTTGTTACGACTTCACC) were used to sequence the whole 16S rRNA gene *via* Sanger sequencing. We designed primer 1492-capro-R using the 16S rRNA gene sequence of *C. galactitolivorans* (National Center for Biotechnology Information, NCBI, FJ805840) as reference. PCR products were sent for sequencing to the Genome Center at the MPI for Developmental Biology (Tübingen, Germany). We used Geneious Prime® 2019.1.3^1^ to trim and align the DNA sequences, using the global Geneious alignment tool at a 93% similarity with gap open and gap extension penalties of 8 and 2, respectively, and 15 refinement iterations. We compared the assembled 16S rRNA gene sequence to the four sequences extracted from the genome using the basic local alignment search tool (BLAST) from the NCBI^2^. We used the most similar sequence (1517 bp) to the Sanger assembly (99.46%) to construct a phylogenetic tree of strain 7D4C2 and its closest relatives. For this, we aligned the 16S rRNA gene sequence to sequences in the Standard nucleotide collection (nr/nt) database using the NCBI BLAST. Because the complete 16S rRNA gene sequence of *Clostridium* sp. W14A (Ransom-Jones and McDonald, 2016) was not available on the NCBI Nucleotide Database, we annotated its genome using Prokka (v. 1.14.5) (Seemann, 2014) (in default settings) and extracted the 16S rRNA gene. We constructed the phylogenetic tree using the Single-Genes-Tree tool^3^. Pairwise sequence similarities between the 16S rRNA gene and closest relatives were calculated using the method recommended by Meier-Kolthoff et al. (2013b) for the 16S rRNA gene sequence available *via* the genome to genome distance calculator (GGDC) web server (Meier-Kolthoff et al., 2013a) accessible at http://ggdc.dsmz.de/. Phylogenies were inferred by the GGDC web server (Meier-Kolthoff et al., 2013a), using the DSMZ phylogenomics pipeline (Meier-Kolthoff et al., 2014), which was adapted to single genes. A multiple-sequence alignment was created with MUSCLE (Edgar, 2004). Maximum likelihood (ML) and maximum parsimony (MP) trees were inferred from the alignment with RAxML (Stamatakis, 2014) and TNT (Goloboff et al., 2008), respectively. For ML, rapid bootstrapping in conjunction with the autoMRE bootstrapping criterion (Pattengale et al., 2010) and subsequent search for the best tree was used. For MP, 1000 bootstrapping replicates were used in conjunction with tree-bisection-and-reconnection branch swapping and ten random sequence addition replicates. The sequences were checked for a compositional bias using the *X*^2^ test as implemented in PAUP^∗^ (Swofford, 2002).

### Genome Sequencing, Assembly, Alignment, and Annotations

The DNA was extracted using a NucleoSpin® Microbial DNA Kit (Macherey-Nagel, Düren, Deutschland), according to the manufacturer’s protocol. The DNA library was prepared using a Rapid barcoding kit (SQK-RBK004, Oxford Nanopore Technologies Ltd., Oxford Science Park, United Kingdom). The DNA was sequenced using a MinION sequencer (Oxford Nanopore Technologies Ltd., Oxford Science Park, United Kingdom) with a single R9.4.1 flow cell. The basecalling was performed with guppy (v 3.6.0) in high accuracy mode. The basecalled reads were assembled using Unicycler (Wick et al., 2017) (v 0.4.8). The assembly was error-corrected for the systematic errors of Nanopore sequencing with three rounds of Racon (Vaser et al., 2017) (v 1.4.10) polishing, as part of the Unicycler pipeline, and an additional round of medaka (v 1.0.1^4^) correction in r941_min_high_g360 mode. The error-corrected assembly resulted in a single, circular, closed chromosome. The quality of the assembly (contamination and completeness) was assessed using CheckM in lineage_wf mode (Parks et al., 2015). We annotated the assembled chromosome using PGAP (Tatusova et al., 2016) (v 2020-03-30.build4489). We obtained 3914 genes in total. The products of the 722 of the 3633 (19.9%) CDS were annotated as “hypothetical protein.” We aligned the predicted CDSs against EggNOG 5.0 (Huerta-Cepas et al., 2019) database, using eggnog-mapper (Huerta-Cepas et al., 2017) (v 2.0.1) with DIAMOND as the choice of the aligner, and assigned a COG annotation to 3338 of them (91.8%).

### Taxonomic Placement

To assign taxonomy, we extracted the identified 16S rRNA gene sequences and aligned them against the NCBI nucleotide database (NCBI-nt). We aligned the whole chromosome against NCBI-nt using minimap2 (Li, 2018) (in asm20 mode) and against NCBI-nr (protein database) using DIAMOND (Buchfink et al., 2015) (with the –long-reads parameter), and assigned taxonomy to it using MEGAN-LR (Huson et al., 2018) (with parameters –lcaCoveragePercent 51 and –longReads). We also used GTDB-Tk (Chaumeil et al., 2020) to classify the genome using the r95 version of the Genome Taxonomy Database (GTDB) (Parks et al., 2018). All methods agreed on assigning strain 7D4C2 to the unclassified microbe *Clostridium* sp. W14A. To further explore the taxonomy of strain 7D4C2, we calculated its ANI using JSpeciesWS (Richter et al., 2016) to all genomes available for the Clostridiales class in GenBank (8662 genomes, accessed on 07/11/2019). We chose the 13 most similar classified microbes for further analysis and used *C. kluyveri* as an outgroup. Next, we compared the percentage of conversed proteins (POCP) as proposed in Qin et al. (2014), and the genome relatedness index as proposed in Barco et al. (2020).

### Phylogenetic Analysis and Synteny of the Genes in the rBOX Cluster

We aligned the genes from strain 7D4C2 that are known to be responsible for chain elongation (i.e., *thl*, *hbd*, *crt*, *acdh*, and *etf-*α and *-*β) against the protein sets of closely related microbes, using DIAMOND (Buchfink et al., 2015) (more-sensitive setting) in BLASTP mode. We obtained the homologs of these proteins in the genomes of bacteria closely related to strain 7D4C2 by filtering DIAMOND hits that cover more than 90% of the query and have more than 45% of positives in the alignment. Because some bacteria had several genes coding for rBOX proteins, for our phylogenetic analyses we focused on the genes that formed a cluster or on those most similar to the genes considered from other bacteria. We computed multiple sequence alignments of the rBOX homologs using MUSCLE (Edgar, 2004) and phylogenetic trees using RAxML (Stamatakis, 2014) with 1000 rounds of bootstrapping (PROTGAMMAAUTO model, parsimony seed set to 12345). We also generated a consensus tree using SplitsTree 5 (v 5.0.0_alpha, with Consensus = Greedy option) (Huson, 1998) of all of the 17 taxa and 6 gene trees. We traced back the genomic coordinates of the rBOX homologs from their annotations on NCBI RefSeq, and used this information to check for synteny and their organization in the genomes manually.

## Results and Discussion

### Strain 7D4C2 Is a Chain-Elongating Bacterium That Converts Sugars Into *n*-Caproate, Lactate, and H_2_ at Mildly Acidic pH

**FIGURE 2:**
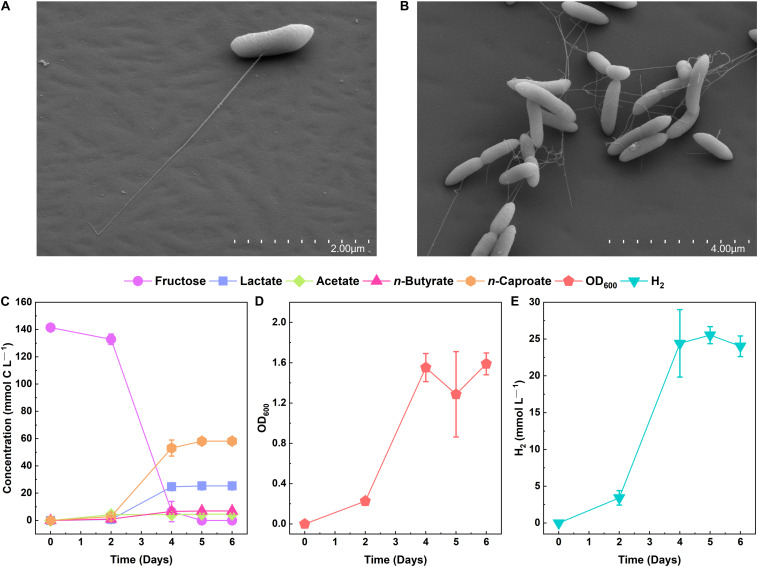
Growth of strain 7D4C2 with fructose at pH 5.5 and 30°C: **(A,B)** scanning electron micrographs of strain 7D4C2; **(C)** fructose conversion into *n*-caproate and lactate; **(D)** growth measured by OD_600_; and **(E)** H_2_ production. Error bars represent one standard deviation among triplicate cultures.

We cryogenically preserved a sample from an open-culture, chain-elongating bioreactor that was operated at a pH of 5.5 and 30°C and fed with ethanol and acetate in our previous laboratory at Cornell University in Ithaca, NY, United States (Spirito, Angenent et al., unpublished work). We revived the sample with ethanol (40 mM), acetate (4 mM), *n*-caproate (4 mM), and *n*-caprylate (4 mM) in basal medium that was buffered with 91.5 mM MES and supplemented with 0.05% w/v yeast extract and vitamins (Supplementary Figure 1). To isolate chain-elongating bacteria, we serially diluted the culture and picked single colonies (pH 5.2, 30°C). Next, the selected colonies were cultured in a liquid medium and further diluted for purification. Since this liquid culture did not consume ethanol, we continued the purification process with fructose as the primary electron donor. The high concentration of MES, the mildly acidic pH (5.2), as well as the added fructose and electron acceptors (*n*-butyrate, *n*-caproate, and *n*-caprylate), inflicted strong selective pressures that allowed the relatively fast isolation (Supplementary Figure 1). Ultimately, the isolate that produced *n*-caproate and showed 100% purity is referred to as strain 7D4C2 (DSM 110548). Strain 7D4C2 is a Gram-positive bacterium (Supplementary Figure 2) and rod-shaped (Figures 2A,B), which produces lactate, acetate, *n*-butyrate, *n*-caproate, biomass, and H_2_ from hexoses at a pH of 5.5 (Figures 2C–E). CO_2_ is also produced (data not shown).

### The Presence of Different Electron Acceptors From 2 to 6 Carbons Influenced Chain Elongation by Strain 7D4C2

**FIGURE 3:**
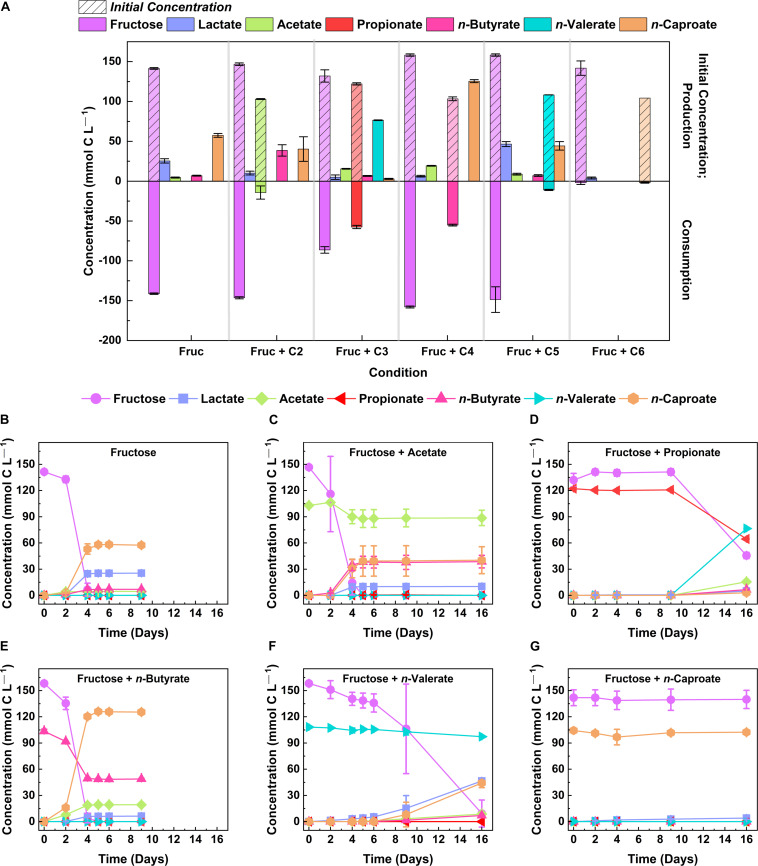
Comparison of lactate and MCCs (i.e., *n*-valerate and *n*-caproate) produced by strain 7D4C2 from fructose and different electron acceptors (C2 → C6): **(A)** comparison of final products and fructose and electron donor consumption among experiments; and **(B–G)** fructose, electron acceptor, and products concentrations throughout the culturing period for each electron acceptor (acetate, propionate, *n*-butyrate, *n*-valerate, and *n*-caproate, respectively). Fruc, fructose; C2, acetate; C3, propionate; C4, *n*-butyrate; C5, *n*-valerate; and C6, *n*-caproate. The initial fructose concentration was 146.4 ± 10.3 mmol C L^– 1^ and the concentration of the electron acceptors was 108.2 ± 8.0 mmol C L^– 1^. The pH value of the test was 5.5 ± 0.02. Error bars represent one standard deviation among triplicate cultures.

Short-chain carboxylates are commonly used as electron acceptors in chain elongation (Jeon et al., 2016; Wang et al., 2018). To study whether strain 7D4C2 was capable of utilizing even- and odd-chain electron acceptors, we grew the isolate at a temperature of 30°C and a pH of 5.5 with fructose (146.4 ± 10.3 mmol C L^–1^) and different carboxylates (108.2 ± 8.0 mmol C L^–1^) from 2 to 6 carbons (i.e., acetate, propionate, *n*-butyrate, *n*-valerate, and *n*-caproate) in separate serum bottles. For the control cultures (fructose without an electron acceptor), strain 7D4C2 achieved a final average concentration of 6.9 ± 0.6 mmol C L^–1^ for *n*-butyrate and 57.5 ± 2.4 mmol C L^–1^ for *n*-caproate (Figures 3A,B), with an *n*-caproate specificity of 60.9 ± 1.5% (i.e., products *per* other carboxylates produced) (Supplementary Table 2). The presence of electron acceptors influenced the metabolism of strain 7D4C2. For acetate as the electron acceptor (13.8 ± 8.1% uptake), the final average *n*-butyrate concentration was higher than the control cultures (38.7 ± 7.2 mmol C L^–1^), while the *n*-caproate concentration was lower (40.3 ± 15.4 mmol C L^–1^), with an *n*-caproate specificity of 44.1 ± 5.9% (Figures 3A,C and Supplementary Table 2). For propionate as the electron acceptor, the 47.1 ± 1.7% uptake changed the metabolism from *n*-caproate to *n*-valerate production (compared to the control cultures) to reach a final average *n*-valerate concentration of 76.5 ± 0.4 mmol C L^–1^, although with a longer lag phase for fructose uptake and biomass production (Figures 3A,D and Supplementary Figure 3). This resulted in an *n*-caproate specificity of only 2.79 ± 0.5% (Supplementary Table 2). Strain 7D4C2 achieved a higher *n*-caproate concentration for *n*-butyrate as the electron acceptor (53.3 ± 1.1% uptake) than for the control and the rest of carboxylates as electron acceptors, resulting in a total average concentration of 125.5 ± 1.9 mmol C L^–1^ and an *n*-caproate specificity of 83.1 ± 44% (Figure 3A and Supplementary Table 2). Previous studies with other chain-elongating bacteria have also observed the highest *n*-caproate specificities with *n*-butyrate (Jeon et al., 2016; Zhu et al., 2017). Moreover, the mmol-C ratio of produced *n*-caproate to lactate was higher at 20:1 for the 7D4C2 cultures with *n*-butyrate than at 2:1 for the control cultures (Figures 3A,E and Supplementary Table 2). For *n*-valerate as the electron acceptor (10.1 ± 0.7% uptake), the final average lactate concentration was higher than the rest of the conditions (46.2 ± 3.2 mmol C L^–1^), and equivalent to the final average *n*-caproate concentration (44.3 ± 5.3 mmol C L^–1^), with an *n*-caproate specificity of 41.4 ± 3.3% (Figures 3A,F and Supplementary Table 2). The presence of this electron acceptor delayed the exponential growth phase for fructose consumption and biomass production (Figures 3A,F and Supplementary Figure 3). We do not completely understand the reasons for these shifts in metabolism but know from theoretical calculations that the ratio of electron donor and electron acceptor has a large thermodynamic effect on product formation (Angenent et al., 2016). Lastly, for *n*-caproate as the electron acceptor, the initial total concentration of 102.4 ± 0.5 mmol C L^–1^ resulted in an undissociated *n*-caproic acid concentration of ∼19.8 mmol C L^–1^ (∼3.3 mM) at a pH value of 5.5, which completely inhibited the metabolism of strain 7D4C2 (Figures 3A,G, Supplementary Figure 3, and Supplementary Table 2). A higher undissociated *n*-caproate concentration was achieved with *n*-butyrate as the electron acceptor (∼24 mmol C L^–1^ or ∼4 mM). However, this concentration was achieved when the OD_600_ reached 1.69 ± 0.06 (Supplementary Figure 3), after all the fructose was consumed (Figure 3E).

### The Specificity of *n*-Caproate Production Was Higher at Mildly Acidic pH Values While That of Lactate Was Higher at Alkaline pH Levels

**FIGURE 4:**
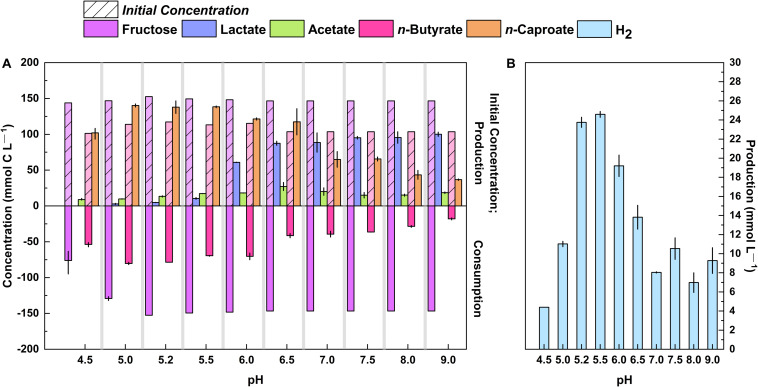
Production of lactate, *n*-caproate, and H_2_ by strain 7D4C2 across a wide pH range (4.5 to 9.0*): **(A)** comparison of final products (lactate, acetate, and *n*-caproate) and fructose and *n*-butyrate consumption among experiments at different pH values; and **(B)** comparison of final H_2_ production among experiments at different pH values. Bars represent minimum and maximum values between duplicate cultures. The initial concentrations of fructose and *n*-butyrate are shown in transparency with a lined pattern. *Initial pH values in an MES-buffered system. The lag phase at pH values of 4.5 and 5.0 was slower than the rest of the experiments (see Supplementary Figure 5).

Next, we investigated lactate and *n*-caproate production of strain 7D4C2 at a pH gradient: from mildly acidic to alkaline pH levels. For this, we cultured strain 7D4C2 at 30°C with a mixture of fructose (148.2 ± 3.2 mmol C L^–1^) and *n*-butyrate (112.2 ± 6.3 mmol C L^–1^) as the substrate at different initial pH values from 4.5 to 9.0 (Figure 4). We did not manually adjust the pH during the culture period, but we strongly buffered the 7D4C2 cultures with 91.5 mM MES. The initial mildly acidic pH values from 4.5 to 5.5 favored the mmol-C ratio of produced *n*-caproate to lactate (lactate below detection at a pH value of 4.5 and 13:1 mmol C L^–1^ at a pH value of 5.5), with final average *n*-caproate concentrations of 93.2 to 146.7 mmol C L^–1^ (Figure 4A). The average *n*-caproate specificities for pH 4.5 to 5.2 were ∼90%, but the specificity decreased to ∼83% for the pH 5.5 condition (Supplementary Table 3). At initial pH values higher than 6.0, the mmol-C ratio of produced *n*-caproate to lactate gradually decreased to 0.4:1 at a pH value of 9.0. Strain 7D4C2 achieved a maximum average lactate concentration of 103.0 mmol C L^–1^ at a pH of 9.0 (Supplementary Table 3). In addition, strain 7D4C2 metabolized less and less *n*-butyrate across the increasing pH gradient (Figure 4A). Together, the changes in metabolism across the alkaline pH values led to a decrease in the final average *n*-caproate concentration from <76.0 to ∼36.0 mmol C L^–1^ for pH 7.0 to 9.0 (Figure 4A), resulting in a decrease in specificity from 37 to 23% (Supplementary Table 3). The H_2_ production in mmol L^–1^ did not follow the exact same trend of *n*-caproate specificity, but it was the highest at the low pH values of 5.2 and 5.5 (Figure 4B). We also cultured strain 7D4C2 at an initial pH of 10.0, but it did not grow (data not shown).

To understand why mildly acidic pH values favored *n*-caproate production, we calculated the Gibbs energy change (Δ*G*_r_) values for fructose and *n*-butyrate conversion into *n*-caproate, H_2_, and CO_2_ by strain 7D4C2 at every pH value studied (4.5–9.0). For this, we used the equations described in Rittmann and McCarty (2001) and the product and fructose information summarized in Supplementary Table 3. As expected, the energy released at mildly acidic pH values was higher than that released at neutral or alkaline pH values (Supplementary Figure 4). Specifically, the Δ*G*_r_ values increased from ∼-61.5 kJ/mol-fructose at a pH value of 4.5 to −29.4 −23.0 kJ/mol-fructose at pH values of 5.0–5.5. From pH values 5.0 to 9.0, the Δ*G*_r_ gradually increased to ∼14.7 kJ/mol-fructose (Supplementary Figure 4). A similar higher energy release from ∼−40 to ∼−50 kJ/mol-substrate was reported by Candry et al. (2020) for the conversion of lactic acid into *n*-caproate by an open culture at decreasing pH values from 7.0 to 4.5. Altogether, these results suggest that chain elongating bacteria that can grow at mildly acidic pH values have a thermodynamic advantage over neutrophilic chain elongating bacteria. However, more research to study the energetics of chain elongation is needed.

### The Optimum pH and Temperature for *n*-Caproate Production Differed for the Growth Rate

As discussed in the previous section, strain 7D4C2 achieved the highest *n*-caproate specificity at mildly acidic pH values (4.5–5.2). However, at a pH of 4.5 and 5.0, the bacterium grew with an extended lag phase compared to the pH values 5.2 and 5.5 (Supplementary Figures 5A,B, 6A–C). Based on the high *n*-caproate specificity (∼88.3%) and concentration (129–147 mmol C L^–1^) in combination with a high growth rate (0.5 d^–1^), the optimum pH value for improved *n*-caproate production was 5.2 (Supplementary Table 3). However, based on biomass production and fructose uptake, this pH value differed from the optimum pH value for growth, which was 6.0. At an initial pH of 6.0, the H_2_ production rate, growth rate (1.3 d^–1^) (Supplementary Figures 5A–D), and fructose consumption rate (37.0 mmol C L^–1^ d^–1^; Supplementary Figures 6A–I and Supplementary Table 3) were the highest for this study, but the strain produced an equivalent mixture of *n*-caproate and lactate (2:1 mmol C L^–1^ in Supplementary Table 3), resulting in a lower *n*-caproate specificity than at a pH of 5.2.

Similar to the experiment with different pH values, we investigated the optimum temperature for *n*-caproate production and growth with strain 7D4C2. For this, we grew the isolate with fructose and *n*-butyrate at different temperatures, ranging from 22.5 to 50°C, and at a pH 6.0 (the optimum pH for growth) in separate serum bottles. We found that strain 7D4C2 achieved a maximum *n*-caproate specificity of ∼67% at a temperature of 30°C (∼107 mmol C L^–1^ in Supplementary Table 3). However, similar to the pH optimum, the optimum temperature for *n*-caproate production differed for the growth rate, which was 37 and 42°C. At these temperatures, the fructose consumption rate was 45.5 mmol C L^–1^ d^–1^, compared to 27.3 mmol C L^–1^ d^–1^ at 30°C, and the H_2_ production rate was the highest (Supplementary Figures 5E,F, 6J–N).

### Product Extraction Increased the *n*-Caproate Selectivity at a pH of 5.2

**FIGURE 5:**
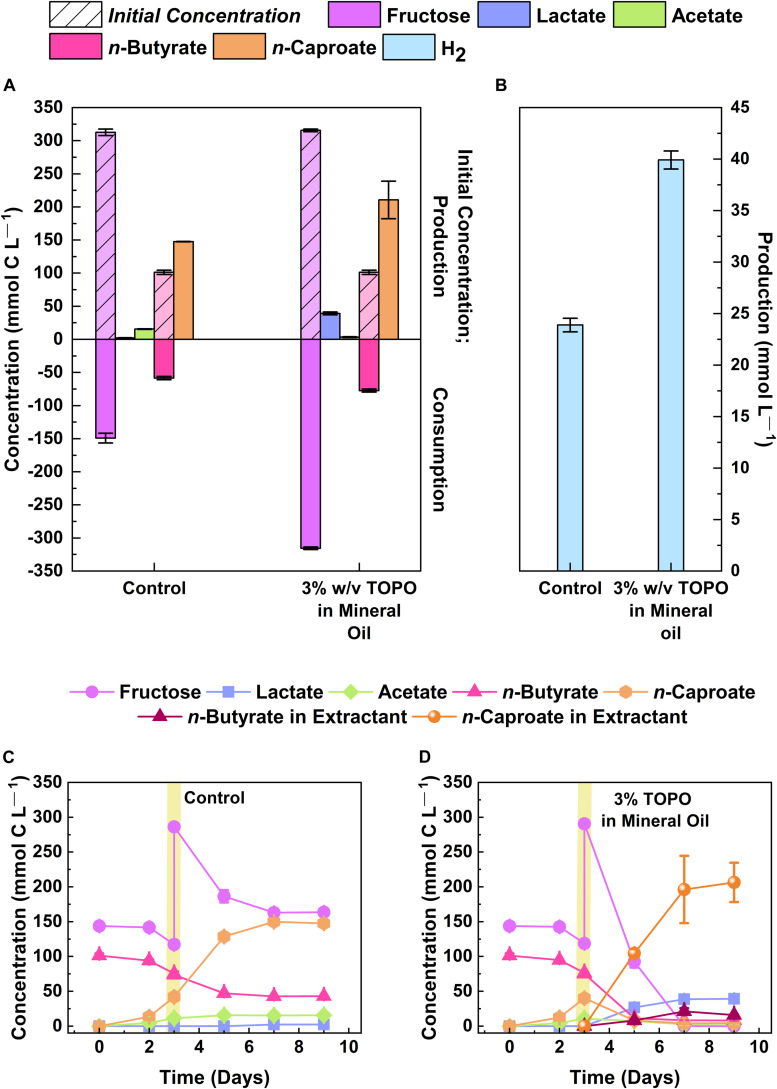
Comparison of *n*-caproate production by strain 7D4C2 with and without product extraction: **(A)** comparison of final products (lactate, acetate, and *n*-caproate) and fructose and *n*-butyrate consumption between experiments with and without mineral oil and 3% w/v TOPO to extract products; **(B)** comparison of final H_2_ production between experiments with and without product extraction; **(C,D)** fructose, *n*-butyrate, and products concentrations throughout the culturing period for the experiments without **(C)** and with product extraction **(D)**. Vertical yellow lines represent the time-point were the fructose was increased (to increase *n*-caproate production) and an equal volume of mineral oil with 3% w/v TOPO was added. Error bars represent one standard deviation among triplicate cultures.

Bioreactors that were operated at mildly acidic pH with in-line product extraction have shown promising MCC production rates and yields (Agler et al., 2014; Ge et al., 2015; Kucek et al., 2016a,b; Spirito et al., 2018). Accordingly, we tested whether strain 7D4C2 could achieve a higher *n*-caproate selectivity (i.e., product *per* substrate fed) when the MCC was extracted during growth, avoiding the toxicity of the undissociated form at mildly acidic pH. For this, we cultured the bacterium with fructose (314.1 ± 2.1 mmol C L^–1^) and *n*-butyrate (101.3 ± 3.2 mmol C L^–1^) as substrates, with product extraction and without product extraction (control) at a pH level of 5.2 and a temperature of 30°C. With the extraction of *n*-caproate, the average concentration of the undissociated MCC in the culture medium remained low at 0.3 ± 0.16 mM, while *n*-caproate production continued until all fructose was depleted by day 7 (Figure 5D and Supplementary Figure 7B). Without extraction, strain 7D4C2 reached the stationary growth phase by day 5 with substrate left over due to inhibition at an undissociated *n*-caproic acid concentration of 4.8 mM (Figures 5A–C and Supplementary Figure 7A). As a result, product extraction of *n*-caproate resulted in a 42.6 ± 19.0% higher *n*-caproate selectivity than the control without extraction (i.e., 62.9 ± 39.7 mmol C L^–1^ more *n*-caproate produced). These results indicate that *Caproiciproducen*s sp. 7D4C2 has the potential as a chain-elongating production bacterium when extraction is desired for sugars as the electron donor.

### Strain 7D4C2 Is Closely Related to Unclassified *Clostridium* sp. W14A, *C. fermentans*, Unclassified *Caproiciproducens* sp. NJN-50, and *C. galactitolivorans*

To assign taxonomy to strain 7D4C2, we sequenced its genome *via* long-read Nanopore sequencing. We obtained 117,171 reads, with an average length of 4,211 bp (N50 of 8,772 bp) and a total size of 486 Mb. The error-corrected genome assembly resulted in a single, circular, and closed chromosome with a full length of 3,947,358 bp and a GC content of 51.6% (RefSeq: GCF_014303955.1). It was annotated with 3,633 protein-coding genes (CDS), 13 rRNA genes (five 5S rRNA genes, four 16S rRNA genes, and four 23S rRNA genes), 60 tRNA genes, 4 ncRNA genes, 1 tmRNA gene, and 203 pseudogenes (154 frameshifted genes). The assembly was 97.85% complete and 1.68% contaminated, according to CheckM (Parks et al., 2015). We aligned the whole genome against the NCBI-nt database. The strain is most similar to four known bacteria: (1) unclassified *Clostridium* sp. W14A (average nucleotide identity, ANI = 97.64; 82.28% aligned bases); (2) *C. fermentans* EA1 (ANI = 97.34; 81.20% aligned bases); (3) unclassified *Caproiciproducens* sp. NJN-50 (ANI = 78.52; 44.36% aligned bases); and (4) *C. galactitolivorans* BS-1 (ANI = 69.65; 28.22% aligned bases) (Supplementary Table 4). Additionally, the genome was classified as the same species as *Clostridium* sp. W14A (genus “UBA1033,” species “UBA1033 sp001695555,” under the family *Acutalibacteraceae*), using the GTDB (Chaumeil et al., 2020). The ANI values for the genome comparison of strain 7D4C2 with *Clostridium* sp. W14A and *C. fermentans* were higher than the cut-off value of 95–96% to define a novel species (∼97.5%; Supplementary Table 4) (Richter and Rosselló-Móra, 2009; Yarza et al., 2014), which indicates that these three bacteria represent different strains of the same species.

To investigate further, we also compared the 16S rRNA gene sequences from strain 7D4C2 with closely related bacteria. We identified four different 16S rRNA gene sequences (1,517–1,524 bp) in the genome of strain 7D4C2, which were 99.03% similar among them. To calculate phylogenetic distances with the other four bacteria, we aligned their 16S rRNA gene sequences (Project ID PRJNA615378) and the Sanger assembly for one of the 16S rRNA gene sequences in strain 7D4C2 (1287 bp, NCBI MT056029) against the NCBI-nt^5^ (accessed January 2020). Since the 16S rRNA gene sequence for *Clostridium* sp. W14A was not publicly available, we annotated the genome for W14A and extracted the 16S rRNA gene sequence. The high-to-low similarities of the 16S rRNA gene sequence for strain 7D4C2 to the four bacteria were in the same order as when the genome alignment was compared: (1) unclassified *Clostridium* sp. W14A (100% similarity to the entire 16S rRNA gene sequence); (2) *C. fermentans* (99.51 ± 0.25% similarity); (3) unclassified *Caproiciproducens* sp. NJN-50 (97.72 ± 0.31%); and (4) *C. galactitolivorans* (94.71 ± 0.35% similarity) (Supplementary Figure 8). A cross comparison for *Clostridium* sp. W14A and *C. fermentans* to *C. galactitolivorans* showed us a 94.83% similarity between *Clostridium* sp. W14A and *C. galactitolivorans*, and a 94.90% similarity between *C. fermentans* and *C. galactitolivorans*, which is slightly outside the quantitative window to group all four strains within a single genus (Yarza et al., 2014). Thus, based on both the genome alignment and 16S rRNA gene sequence comparisons, strain 7D4C2 and its four closest related bacteria are not all strains of the same species, but likely they are all members of the same genus of *Caproiciproducens* spp. This would mean that *C. fermentans* (*Caproicibacter fermentans*) would need to be re-classified as *Caproiciproducens fermentans*.

### The Percentage of Conserved Proteins Also Suggests That Strain 7D4C2, *C. fermentans*, and *C. galactitolivorans* Belong to the Same Genus, but Not the Same Species

**FIGURE 6:**
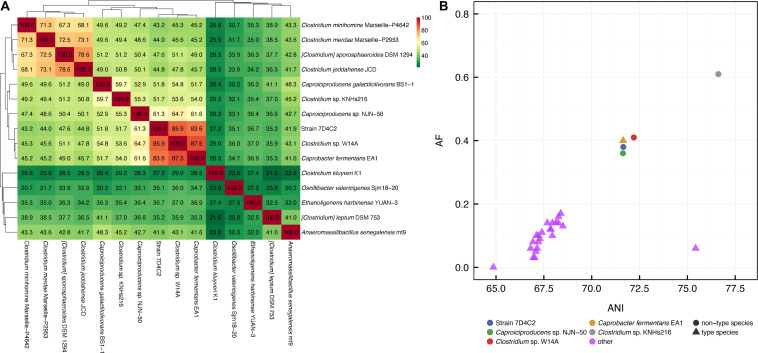
Whole-genome relatedness analyses: **(A)** percentage of conserved proteins (POCP) pairwise values between selected species within the Clostridiales (heterotypic synonym of Eubacteriales). The higher the POCP value (green to red), the closer their evolutionary and genetic distance (Qin et al., 2014). The POCP analysis was performed with genomes publicly available at the NCBI; and **(B)** pairwise ANI (average nucleotide identity) and AF (alignment fraction) values between *C. galactitolivorans* BS-1 and type species (i.e., first accepted species of a genus) of the *Ruminococcaceae* family (heterotypic synonym of Oscillatoriaceae) (magenta), *C. fermentans* EA1 (gold), strain 7D4C2 (red), and three closely related unclassified species (in blue, green, and cyan). The validly published type species information was retrieved from The NamesforLife Database, as suggested in Barco et al. (2020).

To further study whether strain 7D4C2 and its closest related bacteria are members of a single species or a single genus, we calculated the POCP for strain 7D4C2, *C. fermentans*, *C. galactitolivorans*, and their closely related unclassified strains (i.e., *Clostridium* sp. W14A, *Caproiciproducens* sp. NJN-50, and *Clostridium* sp. KNHs216). Besides *Clostridium* sp. KNHs216, we also included additional selected species from the Clostridiales (according to the NCBI Taxonomy Database; heterotypic synonym of Eubacteriales) for this analysis (those with the highest ANI values with strain 7D4C2, Supplementary Table 4). Qin et al., 2014 have suggested that species within the same genus share at least half of their proteins, and therefore their pairwise POCP values are higher than 50% within a clade (Qin et al., 2014). As anticipated from the above results, the pairwise POCP values among strain 7D4C2, *Clostridium* sp. W14A, and *C. fermentans* were high (83.4–87.5%). These three bacteria formed a clade with pairwise POCP values higher than 51.7% with *C. galactitolivorans* and the closely related unclassified strains (i.e., *Caproiciproducens* sp. NJN-50 and *Clostridium* sp. KNHs216), suggesting that all these bacteria belong to the same genus (Qin et al., 2014) (Figure 6A). However, strain 7D4C2, *Clostridium* sp. W14A, *C. fermentans*, and *Caproiciproducens* sp. NJN-50 (POCP: 61.3–87.5%) separated into a different sub-clade from *C. galactitolivorans* and *Clostridium* sp. KNHs216 (POCP: 59.7%) (Figure 6A). The former sub-clade with strain 7D4C2 separated again into two clades with *Caproiciproducens* sp. NJN-50 as the sole strain. Strain 7D4C2, *Clostridium* sp. W14A, and *C. fermentans* are very similar strains and form a separate species based on this analysis and the genome alignment comparison.

In addition, we followed the approach that was suggested by Barco et al. (2020) to demarcate genera based on the relation between genome indices and the distinction of type- and non-type species. We used the ANI of protein-coding genes and the genome AF as considered indices (Barco et al., 2020). For this analysis, we chose *C. galactitolivorans* as a reference bacterium and compared its genome relatedness index (the relation between ANI and AF) to strain 7D4C2, *C. fermentans*, and its three closely related unclassified strains (i.e., *Clostridium* sp. W14A, *Caproiciproducens* sp. NJN-50, and *Clostridium* sp. KNHs216), as well as the type species of each genus within the family *Ruminococcaceae* (according to the NCBI Taxonomy Database; heterotypic synonym of *Oscillatoriaceae*). Results from this analysis supported our other analyses: strain 7D4C2 clustered closely with *Clostridium* sp. W14A, *C. fermentans*, and *Caproiciproducens* sp. NJN-50 (Figure 6B) at higher ANI and AF values than the type species, indicating the similarity to *C. galactitolivorans*. We found still higher ANI and AF values for *Clostridium* sp. KNHs216, which indicates a closer similarity to *C. galactitolivorans* than the other four bacteria (Figure 6B). The clear separation between strain 7D4C2, *C. fermentans*, and the three related unclassified strains from the type species within the *Ruminococcaceae* suggests that neither of these species represents a novel genus, but that they are all members of the *Caproiciproducens*.

### Strain 7D4C2, *C. fermentans*, and *C. galactitolivorans* Belong to the Same Genus Based on Their Phenotype

To further validate that strain 7D4C2, *C. fermentans*, and *C. galactitolivorans* are members of the genus *Caproiciproducens*, we cultured strain 7D4C2, *C. galactitolivorans*, and [*Clostridium*] *leptum* under similar conditions (i.e., complex medium, 37°C, pH of 7.0) and compared the products from glucose fermentation. We chose [*Clostridium*] *leptum* as our reference because it is the closest isolate to *C. galactitolivorans* (Kim et al., 2015), and it is closely related to strain 7D4C2 (Supplementary Figure 8). We then compared our results to those reported for *C. fermentans* EA1 in Flaiz et al. (2020). Both strain 7D4C2 and *C. galactitolivorans* produced lactate, acetate, *n*-butyrate, *n*-caproate, and H_2_/CO_2_, although at different proportions (Supplementary Figure 9). Final average lactate and *n*-caproate concentrations in 7D4C2 cultures were higher than in *C. galactitolivorans* cultures; the lactate concentration was 10.1 ± 0.7 mmol C L^–1^ higher, and the *n*-caproate concentration was 29.1 ± 0.5 mmol C L^–1^ higher (Supplementary Figure 9A). Similarly, the final average *n*-caproate concentration in 7D4C2 cultures was 36.1 ± 1.1 mmol C L^–1^ higher than in C. *galactitolivorans* cultures in a supplemented basal medium at 37°C and a pH of 6.0 (data not shown). [*C*]. *leptum* did not produce lactate nor *n*-caproate, and only ethanol and acetate were detected in the cultures (Supplementary Figure 9A). All three strains produced H_2_, but H_2_ production by *C. galactitolivorans* was the highest (Supplementary Figure 9B). Similar to strain 7D4C2 and *C. galactitolivorans*, *C. fermentans* also produced lactate, acetate, *n*-butyrate, *n*-caproate, and H_2_/CO_2_ from hexoses (Flaiz et al., 2020). The *n*-caproate production per mole of glucose consumed (without the addition of an external electron acceptor) by these three strains was 18–23 g/L for *C. fermentans* (Flaiz et al., 2020) and *C. galactitolivorans*, and ∼40 g/L for strain 7D4C2 at pH 7.0 (Table 1).

**TABLE 1:** Differential characteristics of strain 7D4C2 and closely related species: (1) Strain 7D4C2; (2) *Caproicibacter fermentans* (Flaiz et al., 2020); (3) *Caproiciproducens galactitolivorans* BS-1 (Kim et al., 2015); and (4) [*Clostridium*] *leptum* VPI T7-24-1 (Moore et al., 1976).

Characteristic	1*	2	3	4
Source	Anaerobic reactor	Anaerobic reactor	Anaerobic reactor	Fecal flora
16S rRNA percent identity, %^a^	–	99.51 ± 0.25%	94.71 ± 0.35	93.44 ± 0.14
POCP, %	–	83.4	51.9	35.0
Cell shape	Rod	Rod	Rod	Rod
Cell size (μm)	0.3–0.4 × 1.3–2	0.4–0.8 × 1–3	0.3–0.5 × 2–4	0.6–8.8 × 1.3–2.8
Gram stain	+	−^b^	+	+
Spores observed	No^c^	No^c^	No^c^	Yes
Optimum pH	6.0	7.0	6.5–7.5	NR
Optimum T	37–42	37	40	37
Substrate/products	Glucose, fructose/H_2_, CO_2_, acetate, *n*-butyrate, *n*-caproate, lactate	Fructose/H_2_, CO_2_, acetate, *n*-butyrate, *n*-caproate, lactate, ethanol	Glucose*, galactitol_/_H_2_, CO_2_, acetate, *n*-butyrate, *n*-caproate, ethanol, lactate*	Maltose/H_2_, acetate. Glucose*/H_2_, CO_2_, ethanol*, acetate
*n*-caproate produced/mole of glucose consumed, (g/L)^d^	40.1 ± 0.4 at pH 7.0 47.8 ± 2.9 at pH 5.5	18.3 at pH 7.0	22.7 ± 0.2 at pH 7.0*	0.0*
GC content, %	51.6	51.25	48.1	50.2
Genome length, Mbp	3.95	3.9	2.58	3.27

*Data from this study. NR: not reported.

*^*a*^The 16S rRNA gene percent identity represents an average of the percent identities obtained from the four 16S rRNA gene sequences of strain 7D4C2 extracted from the genome (NCBI PRJNA615378) and the assembly done with Sanger Sequencing (NCBI MT056029).*

*^*b*^Negative staining but cell wall typical of Gram-positive bacteria.*

*^*c*^Spores not observed but the genome encodes one or more sporulation genes.*

*^*d*^n-caproate produced without the addition of an external electron acceptor (e.g., n-butyrate).*

*The genomes of C. fermentans, C. galactitolivorans, and C. leptum were downloaded from the NCBI (accession numbers, NZ_VWXL00000000, SRMQ00000000, and ABCB00000000, respectively). POCP, percentage of conserved proteins with strain 7D4C2.*

To identify phenotypic differences between strain 7D4C2, *C. fermentans*, and *C. galactitolivorans*, we studied the carbohydrate utilization of strain 7D4C2 using the AN MicroPlate^TM^ from Biolog (Hayward, CA, United States) (Supplementary Figure 10) and we compared the results to those reported for *C. fermentans* (Flaiz et al., 2020) and *C. galactitolivorans* (Kim et al., 2015). From the seven carbohydrates compared between strain 7D4C2 and *C. fermentans*, all but glycerol (oxidized by strain 7D4C2 and *C. galactitolivorans*) and D-galactose (oxidized by *C. fermentans* and *C. galactitolivorans*) showed similar utilization (Supplementary Table 5). The carbohydrate utilization by strain 7D4C2 and *C. galactitolivorans* differed in 13 out of 30 carbohydrates compared (Supplementary Table 5). Other differential characteristics between strain 7D4C2, *C. fermentans*, *C. galactitolivorans*, and [*C.*] *leptum* included optimal pH and temperature and genome length (Table 1).

In general, our work shows that strain 7D4C2 and *C. fermentans* have a similar phenotype to *C. galactitolivorans*. Therefore, based on the ∼5% dissimilarity between their 16S rRNA gene sequences and the >51.7% shared conserved proteins, we propose that: (1) strain 7D4C2, the unclassified *Clostridium* sp. W14A, *C. fermentans*, the unclassified *Caproiciproducens* sp. NJN-50, *C. galactitolivorans*, and the unclassified *Clostridium* sp. KNHs216 belong to the genus *Caproiciproducens*; and (2) strain 7D4C2, the unclassified *Clostridium* sp. W14A, and *C. fermentans*, are very similar strains of a new species within the *Caproiciproducens*. We propose *C. fermentans* as the name for these three strains based on the work by Flaiz et al. (2020). *C. fermentans* 7D4C2 produced higher amounts of *n*-caproate than *C. galactitolivorans* and what is reported for *C. fermentans* EA1 (Flaiz et al., 2020) (Supplementary Figure 9A and Table 1). Thus, strain 7D4C2 has the highest potential to treat organic waste in pure culture chain-elongating bioreactors with in-line product extraction. Moreover, the growth of *Caproiciproducens* species and their *n*-caproate production can be improved in open culture bioreactors by selecting mildly acidic pH values, as observed by Candry et al. (2020) in bioreactors treating a synthetic waste stream.

### The Six rBOX Genes in *Caproiciproducens* Species Are Located Next to Each Other, Forming a Gene Cluster

To further study the chain-elongation metabolism of strain 7D4C2, we identified the rBOX genes (*thl*, *hbd*, *crt*, *acdh*, and *etf-*α and *-*β; Figure 1) in its genome and we compared them to those in: (1) closely related bacteria (i.e., the proposed *Caproiciproducens* species); (2) bacteria with similar rBOX genes (i.e., *Anaeromassilibacillus senegalensis*, *Eubacterium limosum*, and several *Clostridium* species); and (3) well known chain-elongating bacteria (i.e., *Clostridium kluyveri*, *Oscillibacter valericigenes*, unclassified *Ruminococcaceae* CPB6, *M. hexanoica*, and *M. elsdenii*). The number of copies for each gene varied from 1 to 14 for the included bacteria (Supplementary Data Sheet 1). The genomes of strain 7D4C2, unclassified *Clostridium* sp. W14A, and *C. fermentans* EA1 have two copies for *thl*, 2-3 copies for *acdh* and *etf-*α, three copies for *etf-*β, and one copy for *hbd* and *crt*. Differently, *Caproiciproducen*s sp. NJN-50 and *Clostridium* sp. KNHs216 encode several copies for each rBOX gene, and *C. galactitolivorans* has only one copy for each gene (Supplementary Data Sheet 1). In general, the genomes of the analyzed bacteria contain multiple copies for some or all of the rBOX genes. However, *C. galactitolivorans*, *A. senegalensis*, and uncultured *Ruminococcaceae* CPB6 only contain a single copy (Supplementary Data Sheet 1).

**FIGURE 7:**
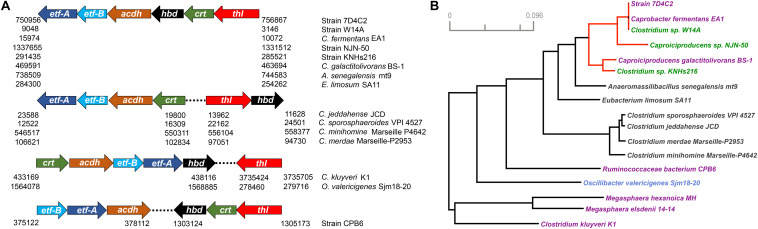
Reverse β-oxidation genes for strain 7D4C2 and bacteria with similar genes, as well as in known *n*-caproate producers: **(A)** position of the rBOX genes that cluster together in these bacteria. The numbers below the arrows indicate the position (base pairs) of the genes for each bacterium on the right column; and **(B)** consensus phylogenetic tree of all 6 rBOX genes that cluster together*. Red lines indicate the *Caproiciproducens* clade. Microbial names highlighted in purple denote *n*-caproate producers, in green are potential *n*-caproate producers, and in blue *n*-valerate producers. The phylogenetic distances of each of the rBOX genes in these bacteria are shown in Supplementary Figure 11. *As the rBOX genes in the *Megasphaera* species do not cluster, for this analysis, we considered the genes most similar to strain 7D4C2.

One copy for each of the rBOX genes (*thl*, *hbd*, *crt*, *acdh*, and *etf-*α and *-*β) in strain 7D4C2 are located next to each other, forming a 5,903-base pair-long gene cluster (Figure 7A). We observed the same synteny of the rBOX cluster for the genomes of bacteria that are closely related to the *Caproiciproducens*. Similarly, this synteny was found for *A. senegalensis*, which is not known as a chain elongator, and *E. limosum*, which is an acetate and *n*-butyrate producer (Roh et al., 2011; Park et al., 2017), and which is capable of *n*-caproate production at high *n*-butyrate concentrations (Lindley et al., 1987) (Figure 7A). In addition, this gene cluster has been identified in novel Clostridia that produce *n*-caproate from lactate (Liu et al., 2020). The arrangement of the rBOX genes varied for other bacteria. For the *Clostridium* species (i.e., *Clostridium jeddahense, Clostridium sporosphaeroides, Clostridium minihomine*, and *Clostridium merdae*), which are not known to produce *n*-caproate, the rBOX gene cluster is separated; *thl* and *hbd* form one cluster and *crt*, *acdh*, *etf-*α, and *etf-*β form a separate cluster, approximately 5 kbp away from each other and on the opposite strand (Figure 7A and Supplementary Data Sheet 1). More work is needed to understand if the rBOX genes in these bacteria are involved in MCC production. For the well-known chain-elongating bacteria *C. kluyveri* and *O. valericigenes* [an *n*-valerate producer (Lino et al., 2007)], their genomes have one copy of five rBOX genes (all but *thl*) in synteny (Figure 7A). The *thl* genes in these two chain-elongating bacteria are separated from the rest of the rBOX genes. The three thiolase genes in *C. kluyveri* form a separate cluster 658,054 bp away from the rBOX cluster (Supplementary Data Sheet 1). In *Ruminococcaceae* bacterium CPB6, *acdh*, *etf-*α, and *etf-*β cluster together, while *thl*, *hbd*, and *crt* cluster further away (924,173 bp) from the first three genes (Figure 7A and Supplementary Data Sheet 1). The rBOX genes of *M. hexanoica* and *M. elsdenii* are not in an apparent synteny, although those of *M. hexanoica*, except *thl*, are close to each other (Supplementary Data Sheet 1). More work is required to understand whether an advantage exists for chain-elongating bacteria with a gene cluster for rBOX genes compared when these genes are located separately on the genome.

### The rBOX Genes in Strain 7D4C2 Are Mostly Similar to Those in *Caproiciproducens* Species and Relatively Distant to Those in Other Chain-Elongating Bacteria

We built individual gene trees with the 6 rBOX genes and a consensus tree out of them in strain 7D4C2, closely related bacteria, bacteria with similar rBOX genes, and known chain-elongating bacteria. As the gene copies varied for different bacteria, we included in the analyses the rBOX genes that are located close to each other (forming a cluster) or that are most similar to those in strain 7D4C2 (Supplementary Data Sheet 1). The analysis showed that the rBOX genes of strain 7D4C2 are identical to those of *Clostridium* sp. W14A and *C. fermentans*. In general, these genes are very similar to those of other members of the POCP clade (i.e., *Caproiciproducen*s sp. NJN-50, *C. galactitolivorans*, and *Clostridium* sp. KNHs216; Figures 6A, 7B). The rBOX genes of strain 7D4C2 are also similar to those of less closely related bacteria, such as *A. senegalensis* and *E. limosum*, but relatively distant to those of other chain-elongating bacteria (i.e., *C. kluyveri*, *O. valericigenes*, *Ruminococcaceae* bacterium CPB6, *M. hexanoica*, and *M. elsdenii*) (Figure 7B).

The individual gene trees showed that the phylogenetic distance between the rBOX genes of strain 7D4C2 and related bacteria varies for each gene. Nonetheless, the rBOX genes of the proposed *Caproiciproducens* spp. are often within a monophyletic clade, and are always close to each other (Supplementary Figure 11). The rBOX genes of *A. senegalensis* and *E. limosum* are phylogenetically closest to those of the *Caproiciproducens*. In the cases of *acdh* and *etf-*β, these bacteria form a cluster together with *Caproiciproducens* species. The exceptions are *thl* and *hbd* in *E. limosum*, which are distant to the *Caproiciproducens* and closer to the *Clostridium* species (Supplementary Figure 11). The lactate consumer *Ruminococcaceae* bacterium CPB6 shows an interesting pattern in the individual gene trees. In the gene trees of *thl* and *crt*, strain CPB6 clusters within the *Caproiciproducens* clade, but it is distant to these bacteria in the rest of the gene trees (Supplementary Figure 11). Because of this, in the consensus tree, strain CPB6 is relatively distant to strain 7D4C2 (Figure 7B). In summary, the distances of the rBOX genes varied among individual gene trees, both within well-known and not known chain-elongating bacteria, showing no consensus on a particular gene being relatively more conserved in chain-elongating bacteria than other bacteria.

## Conclusion

We isolated a chain elongating bacterium (strain 7D4C2) that primarily produces *n*-caproate from carbohydrates at mildly acidic pH values (4.5–5.5). The isolate has the potential to be used in chain-elongating bioreactors that treat organic waste and are operated at mildly acidic pH with in-line product extraction. After extensive comparison of the whole-genomes of strain 7D4C2 with the isolates *C. galactitolivorans* and *C. fermentans*, and closely related unclassified bacteria (*Clostridium* sp. W14A, *Caproiciproducens* sp. NJN-50, and *Clostridium* sp. KNHs216), we would classify strain 7D4C2 and *C. fermentans* into the same genus of *Caproiciproducens* with *C. galactitolivorans*. The comparable phenotype and similar chain-elongation metabolism between strain 7D4C2, *C. fermentans*, and *C. galactitolivorans* also support that these bacteria belong to the same genus. Thus, we name our isolate *C. fermentans* 7D4C2, which is the same species as *Clostridium* sp. W14A and *C. fermentans*. The rBOX genes of these *Caproiciproducens* species are highly similar and relatively distant to the genes of other chain-elongating bacteria. The 6 rBOX genes in the *Caproiciproducens* spp. are located next to each other, forming a gene cluster. This rBOX cluster is also present in bacteria not known to chain elongate, such as *A. senegalensis* and several *Clostridium* spp. The close similarity of the rBOX genes of strain 7D4C2 with these bacteria requires further investigation to understand what defines a chain elongator.

## Data Availability Statement

Strain 7D4C2 was deposited in the German Collection of Microorganisms and Cell Cultures (DSMZ) under the accession number DSM 110548. The datasets generated for this study can be found in online repositories. The names of the repository/repositories and accession number(s) can be found in the article/Supplementary Material.

## Author Contributions

LA conceived the project. SE-E designed and guided the study. MT and SE-E performed the lab experiments. CB performed the bioinformatics analyses. MT, CB, and SE-E analyzed the data and prepared the figures and tables. SE-E, LA, CB, and MT drafted the manuscript. BJ and IB performed the genome sequencing. RW advised on the sequencing tools. LA and DH provided guidance. All authors edited the manuscript and approved the final manuscript.

## Conflict of Interest

The authors declare that the research was conducted in the absence of any commercial or financial relationships that could be construed as a potential conflict of interest.

## Footnotes

1 http://www.geneious.com

2 https://blast.ncbi.nlm.nih.gov/Blast.cgi

3 http://ggdc.dsmz.de/

4 https://github.com/nanoporetech/medaka

5 ftp://ftp.ncbi.nlm.nih.gov/blast/db/FASTA/

## Supplementary Material

Supplementary Data Sheet 1 Location and percent identity of all rBOX genes in strain 7D4C2, closely related bacteria, bacteria with similar rBOX genes, and known chain-elongating bacteria. In green: rBOX genes used in the phylogenetic analyses.

## Abbreviations

MCC: medium-chain carboxylate (comprising both the dissociated and undissociated forms)
SCC: short-chain carboxylate (comprising both the dissociated and undissociated forms)
rBOX: reverse β-oxidation
Thl: thiolase
HBD: 3-hydroxybutyryl-CoA dehydrogenase
Crt: crotonyl-CoA
ACDH: acyl-CoA dehydrogenase
ETF: electron transport flavoprotein
ANI: average nucleotide identity
POCP: percentage of conserved proteins
AF: aligned fraction
OD_600_: optical density measured at 600 nm
Δ*G*_r_: Gibbs energy change.

## References

[B1] Agler M. T. Spirito C. M. Usack J. G. Werner J. J. Angenent L. T. (2012a). Chain elongation with reactor microbiomes: upgrading dilute ethanol to medium-chain carboxylates. *Energy Environ. Sci.* 5:8189. 10.2166/wst.2013.549 24434969

[B2] Agler M. T. Spirito C. M. Usack J. G. Werner J. J. Angenent L. T. (2014). Development of a highly specific and productive process for *n*-caproic acid production: applying lessons from methanogenic microbiomes. *Water Sci. Technol.* 69 62–68. 10.1039/c2ee22101b 24434969

[B3] Agler M. T. Werner J. J. Iten L. B. Dekker A. Cotta M. A. Dien B. S. (2012b). Shaping reactor microbiomes to produce the fuel precursor *n*-butyrate from pretreated cellulosic hydrolysates. *Environ. Sci. Technol.* 46 10229–10238. 10.1021/es302352c 22894772

[B4] Angenent L. T. Richter H. Buckel W. Spirito C. M. Steinbusch K. J. J. Plugge C. M. (2016). Chain elongation with reactor microbiomes: open-culture biotechnology to produce biochemicals. *Environ. Sci. Technol.* 50 2796–2810. 10.1021/acs.est.5b04847 26854969

[B5] Barco R. Garrity G. Scott J. Amend J. Nealson K. Emerson D. (2020). A genus definition for bacteria and archaea based on a standard genome relatedness index. *mBio* 11:e002475-19. 10.1128/mBio.02475-19 PMC6960282 31937639

[B6] Buchfink B. Xie C. Huson D. H. (2015). Fast and sensitive protein alignment using DIAMOND. *Nat. Methods* 12:59. 10.1038/nmeth.3176 25402007

[B7] Candry P. Radić L. Favere J. Carvajal-Arroyo J. M. Rabaey K. Ganigué R. (2020). Mildly acidic pH selects for chain elongation to caproic acid over alternative pathways during lactic acid fermentation. *Water Res.* 186 116396. 10.1016/j.watres.2020.116396 32920334

[B8] Chaumeil P.-A. Mussig A. J. Hugenholtz P. Parks D. H. (2020). *GTDB-Tk: A Toolkit to Classify Genomes With the Genome Taxonomy Database.* Oxford: Oxford University Press, 10.1093/bioinformatics/btz848 PMC7703759 31730192

[B9] Contreras-Dávila C. A. Carrión V. J. Vonk V. R. Buisman C. N. J. Strik D. P. B. T. B. (2020). Consecutive lactate formation and chain elongation to reduce exogenous chemicals input in repeated-batch food waste fermentation. *Water Res.* 169 115215. 10.1016/j.watres.2019.115215 31678751

[B10] Desbois A. P. (2012). Potential applications of antimicrobial fatty acids in medicine, agriculture and other industries. *Recent Pat. Antiinfect. Drug Discov.* 7 111–122. 10.2174/157489112801619728 22630821

[B11] Duber A. Jaroszynski L. Zagrodnik R. Chwialkowska J. Juzwa W. Ciesielski S. (2018). Exploiting the real wastewater potential for resource recovery – n-caproate production from acid whey. *Green Chem.* 20 3790–3803. 10.1039/C8GC01759J

[B12] Edgar R. C. (2004). MUSCLE: multiple sequence alignment with high accuracy and high throughput. *Nucl. Acids Res.* 32 1792–1797. 10.1093/nar/gkh340 15034147 PMC390337

[B13] Esquivel-Elizondo S. Bağcı C. Temovska M. Jeon B. S. Bessarab I. Williams R. B. H. (2020). The isolate *Caproiciproducens* sp. 7D4C2 produces *n*-caproate at mildly acidic conditions from hexoses: genome and rBOX comparison with related strains and chain-elongating bacteria. *bioRxiv[Preprint].* 10.1101/2020.07.19.210914 PMC7873966 33584563

[B14] Flaiz M. Baur T. Brahner S. Poehlein A. Daniel R. Bengelsdorf F. R. (2020). *Caproicibacter fermentans* gen. nov., sp. nov., a new caproate-producing bacterium and emended description of the genus *Caproiciproducens*. *Int. J. Syst. Evol. Microbiol.* 70 5575–5599. 10.1099/ijsem.0.004283 32584751

[B15] Ge S. Usack J. G. Spirito C. M. Angenent L. T. (2015). Long-term *n*-caproic acid production from yeast-fermentation beer in an anaerobic bioreactor with continuous product extraction. *Environ. Sci. Technol.* 49 8012–8021. 10.1021/acs.est.5b00238 25941741

[B16] Goloboff P. A. Farris J. S. Nixon K. C. (2008). TNT, a free program for phylogenetic analysis. *Cladistics* 24 774–786. 10.1111/j.1096-0031.2008.00217.x

[B17] Harroff L. A. Liotta J. L. Bowman D. D. Angenent L. T. (2017). Inactivation of *Ascaris* eggs in human fecal material through *in situ* production of carboxylic acids. *Environ. Sci. Technol.* 51 9729–9738. 10.1021/acs.est.7b02014 28759229

[B18] Harvey B. G. Meylemans H. A. (2014). 1-Hexene: a renewable C6 platform for full-performance jet and diesel fuels. *Green Chem.* 16 770–776. 10.1039/c3gc41554f

[B19] Huerta-Cepas J. Forslund K. Coelho L. P. Szklarczyk D. Jensen L. J. Von Mering C. (2017). Fast genome-wide functional annotation through orthology assignment by eggNOG-mapper. *Mol. Biol. Evol.* 34 2115–2122. 10.1093/molbev/msx148 28460117 PMC5850834

[B20] Huerta-Cepas J. Szklarczyk D. Heller D. Hernández-Plaza A. Forslund S. K. Cook H. (2019). eggNOG 5.0: a hierarchical, functionally and phylogenetically annotated orthology resource based on 5090 organisms and 2502 viruses. *Nucl. Acids Res‘* 47 D309–D314. 10.1093/nar/gky1085 30418610 PMC6324079

[B21] Huson D. H. (1998). SplitsTree: analyzing and visualizing evolutionary data. *Bioinformatics* 14 68–73. 10.1093/bioinformatics/14.1.68 9520503

[B22] Huson D. H. Albrecht B. Bağcı C. Bessarab I. Gorska A. Jolic D. (2018). MEGAN-LR: new algorithms allow accurate binning and easy interactive exploration of metagenomic long reads and contigs. *Biol. Direct* 13:6. 10.1186/s13062-018-0208-7 29678199 PMC5910613

[B23] Jeon B. S. Choi O. Um Y. Sang B.-I. (2016). Production of medium-chain carboxylic acids by *Megasphaera* sp. MH with supplemental electron acceptors. *Biotechnol. Biofuels* 9:129. 10.1186/s13068-016-0549-3 27340431 PMC4918077

[B24] Jeon B. S. Kim B.-C. Um Y. Sang B.-I. (2010). Production of hexanoic acid from D-galactitol by a newly isolated *Clostridium* sp. *BS-*1. *Appl. Microbiol. Biotechnol.* 88 1161–1167. 10.1007/s00253-010-2827-5 20721546

[B25] Jeon B. S. Moon C. Kim B.-C. Kim H. Um Y. Sang B.-I. (2013). *In situ* extractive fermentation for the production of hexanoic acid from galactitol by *Clostridium* sp. BS-1. *Enzyme Microb. Tech.* 53 143–151. 10.1016/j.enzmictec.2013.02.008 23830453

[B26] Kenealy W. R. Cao Y. Weimer P. J. (1995). Production of caproic acid by cocultures of ruminal cellulolytic bacteria and *Clostridium kluyveri* grown on cellulose and ethanol. *Appl. Microbiol. Biotechnol.* 44 507–513. 10.1007/BF00169952 8597554

[B27] Kim B.-C. Seung Jeon B. Kim S. Kim H. Um Y. Sang B.-I. (2015). *Caproiciproducens galactitolivorans* gen. nov., sp. nov., a bacterium capable of producing caproic acid from galactitol, isolated from a wastewater treatment plant. *Int. J. Syst. Evol. Microbiol.* 65 4902–4908. 10.1099/ijsem.0.000665 26474980

[B28] Klask C.-M. Kliem-Kuster N. Molitor B. Angenent L. T. (2020). Nitrate feed improves growth and ethanol production of *Clostridium ljungdahlii* with CO2 and H2, but results in stochastic inhibition events. *Front. Microbiol.* 11:724. 10.3389/fmicb.2020.00724 32435236 PMC7219301

[B29] Kucek L. A. Nguyen M. Angenent L. T. (2016a). Conversion of L-lactate into *n*-caproate by a continuously fed reactor microbiome. *Water Res.* 93 163–171. 10.1016/j.watres.2016.02.018 26905795

[B30] Kucek L. A. Spirito C. M. Angenent L. T. (2016b). High *n*-caprylate productivities and specificities from dilute ethanol and acetate: chain elongation with microbiomes to upgrade products from syngas fermentation. *Energy Environ. Sci.* 9 3482–3494. 10.1039/c6ee01487a

[B31] Lanjekar V. B. Marathe N. P. Ramana V. V. Shouche Y. S. Ranade D. R. (2014). *Megasphaera indica* sp. nov., an obligate anaerobic bacteria isolated from human faeces. *Int. J. Syst. Evol. Microbiol* 64(Pt. 7) 2250–2256. 10.1099/ijs.0.059816-0 24711592

[B32] Levy P. F. Sanderson J. E. Kispert R. G. Wise D. L. (1981). Biorefining of biomass to liquid fuels and organic chemicals. *Enzyme Microb. Techn.* 3 207–215. 10.1016/0141-0229(81)90087-9

[B33] Li H. (2018). Minimap2: pairwise alignment for nucleotide sequences. *Bioinformatics* 34 3094–3100. 10.1093/bioinformatics/bty191 29750242 PMC6137996

[B34] Lindley N. Loubiere P. Pacaud S. Mariotto C. Goma G. (1987). Novel products of the acidogenic fermentation of methanol during growth of *Eubacterium limosum* in the presence of high concentrations of organic acids. *Microbiology* 133 3557–3563. 10.1099/00221287-133-12-3557

[B35] Lino T. Mori K. Tanaka K. Suzuki K.-I. Harayama S. (2007). *Oscillibacter valericigenes* gen. nov., sp. nov., a valerate-producing anaerobic bacterium isolated from the alimentary canal of a Japanese corbicula clam. *ıInt. J. Syst. Evol. Microbiol* 57 1840–1845. 10.1099/ijs.0.64717-0 17684268

[B36] Liu B. Popp D. Sträuber H. Harms H. Kleinsteuber S. (2020). Three novel clostridia isolates produce *n*-caproate and iso-butyrate from lactate: Comparative genomics of chain-elongating bacteria. *Microorganisms* 8 10.3390/microorganisms8121970 PMC7764203 33322390

[B37] Marounek M. Fliegrova K. Bartos S. (1989). Metabolism and some characteristics of ruminal strains of *Megasphaera elsdenii*. *Appl. Environ. Microbiol.* 55 1570–1573. 10.1128/aem.55.6.1570-1573.1989 2764566 PMC202906

[B38] Meier-Kolthoff J. P. Auch A. F. Klenk H.-P. Göker M. (2013a). Genome sequence-based species delimitation with confidence intervals and improved distance functions. *BMC Bioinform.* 14:60. 10.1186/1471-2105-14-60 23432962 PMC3665452

[B39] Meier-Kolthoff J. P. Göker M. Spröer C. Klenk H.-P. (2013b). When should a DDH experiment be mandatory in microbial taxonomy? *Arch. Microbiol.* 195 413–418. 10.1007/s00203-013-0888-4 23591456

[B40] Meier-Kolthoff J. P. Hahnke R. L. Petersen J. Scheuner C. Michael V. Fiebig A. (2014). Complete genome sequence of DSM 30083T, the type strain (U5/41T) of *Escherichia coli*, and a proposal for delineating subspecies in microbial taxonomy. *Stand. Genomic Sci.* 9:2. 10.1186/1944-3277-9-2 25780495 PMC4334874

[B41] Moore W. E. C. Johnson J. L. Holdeman L. V. (1976). Emendation of *Bacteroidaceae* and *Butyrivibrio* and descriptions of *Desulfomonas* gen. nov. and ten new species in the genera *Desulfomonas, Butyrivibrio, Eubacterium, Clostridium*, and *Ruminococcus*. *Int. J. Syst. Evol. Microbiol.* 26 238–252. 10.1099/00207713-26-2-238

[B42] Park S. Yasin M. Jeong J. Cha M. Kang H. Jang N. (2017). Acetate-assisted increase of butyrate production by *Eubacterium limosum* KIST612 during carbon monoxide fermentation. *Biores. Technol.* 245 560–566. 10.1016/j.biortech.2017.08.132 28898856

[B43] Parks D. H. Chuvochina M. Waite D. W. Rinke C. Skarshewski A. Chaumeil P.-A. (2018). A standardized bacterial taxonomy based on genome phylogeny substantially revises the tree of life. *Nat. Biotechnol.* 36 996–1004. 10.1038/nbt.4229 30148503

[B44] Parks D. H. Imelfort M. Skennerton C. T. Hugenholtz P. Tyson G. W. (2015). CheckM: assessing the quality of microbial genomes recovered from isolates, single cells, and metagenomes. *Genome Res.* 25 1043–1055. 10.1101/gr.186072.114 25977477 PMC4484387

[B45] Pattengale N. D. Alipour M. Bininda-Emonds O. R. P. Moret B. M. E. Stamatakis A. (2010). How many bootstrap replicates are necessary? *J. Comp. Biol.* 17 337–354. 10.1089/cmb.2009.0179 20377449

[B46] Qin Q.-L. Xie B.-B. Zhang X.-Y. Chen X.-L. Zhou B.-C. Zhou J. (2014). A proposed genus boundary for the prokaryotes based on genomic insights. *J. Bacteriol.* 196 2210–2215. 10.1128/JB.01688-14 24706738 PMC4054180

[B47] Ransom-Jones E. McDonald J. E. (2016). Draft Genome Sequence of *Clostridium* sp. *strain W*14A Isolated from a cellulose-degrading biofilm in a landfill leachate microcosm. *Genome Announc.* 4:e00985-16 10.1128/genomeA.00985-1 PMC5034129 27660778

[B48] Richter M. Rosselló-Móra R. (2009). Shifting the genomic gold standard for the prokaryotic species definition. *Proc. Natl. Acad. Sci. U.S.A.* 106 19126–19131. 10.1073/pnas.0906412106 19855009 PMC2776425

[B49] Richter M. Rosselló-Móra R. Oliver Glöckner F. Peplies J. (2016). JSpeciesWS: a web server for prokaryotic species circumscription based on pairwise genome comparison. *Bioinformatics* 32 929–931. 10.1093/bioinformatics/btv681 26576653 PMC5939971

[B50] Rittmann B. E. McCarty P. L. (2001). *Environmental Biotechnology: Principles and Applications.* New York, NY: McGraw-Hill.

[B51] Roddick F. A. Britz M. L. (1997). Production of hexanoic acid by free and immobilised cells of *Megasphaera elsdenii*: Influence of *in-situ* product removal using ion exchange resin. *J. Chem. Tech. Biotechnol.* 69 383–391. 10.1002/(sici)1097-4660(199707)69:3<383::aid-jctb723>3.0.co;2-h

[B52] Roh H. Ko H.-J. Kim D. Choi D. G. Park S. Kim S. (2011). Complete genome sequence of a carbon monoxide-utilizing acetogen, *Eubacterium limosum* KIST612. *J. Bacteriol.* 193 307–308. 10.1128/JB.01217-10 21036996 PMC3019962

[B53] Ruaud A. Esquivel-Elizondo S. de la Cuesta-Zuluaga J. Waters J. L. Angenent L. T. Youngblut N. D. (2020). Syntrophy *via* interspecies H2 transfer between *Christensenella* and *Methanobrevibacter* underlies their global cooccurrence in the human gut. *mBio* 11 e003235-19. 10.1128/mBio.03235-19 32019803 PMC7002349

[B54] Russell J. (1992). Another explanation for the toxicity of fermentation acids at low pH: anion accumulation versus uncoupling. *J. Appl. Bacteriol.* 73 363–370. 10.1111/j.1365-2672.1992.tb04990.x

[B55] Seemann T. (2014). Prokka: rapid prokaryotic genome annotation. *Bioinformatics* 30 2068–2069. 10.1093/bioinformatics/btu153 24642063

[B56] Spirito C. M. Marzilli A. M. Angenent L. T. (2018). Higher substrate ratios of ethanol to acetate steered chain elongation towards *n*-caprylate in a bioreactor with product extraction. *Environ. Sci. Technol.* 52 13438–13447. 10.1021/acs.est.8b03856 30335369

[B57] Spirito C. M. Richter H. Rabaey K. Stams A. J. M. Angenent L. T. (2014). Chain elongation in anaerobic reactor microbiomes to recover resources from waste. *Curr. Opin. Biotechnol.* 27 115–122. 10.1016/j.copbio.2014.01.003 24487179

[B58] Stamatakis A. (2014). RAxML version 8: a tool for phylogenetic analysis and post-analysis of large phylogenies. *Bioinformatics* 30 1312–1313. 10.1093/bioinformatics/btu033 24451623 PMC3998144

[B59] Swofford D. L. (2002). *PAUP^∗^: Phylogenetic Analysis Using Parsimony (^∗^and other methods). 4.0. B5.* Sunderland: Sinauer Associates.

[B60] Tatusova T. DiCuccio M. Badretdin A. Chetvernin V. Nawrocki E. P. Zaslavsky L. (2016). NCBI prokaryotic genome annotation pipeline. *Nucl. Acids Res.* 44 6614–6624. 10.1093/nar/gkw569 27342282 PMC5001611

[B61] Vaser R. Sović I. Nagarajan N. Šikić M. (2017). Fast and accurate *de novo* genome assembly from long uncorrected reads. *Genome Res.* 27 737–746. 10.1101/gr.214270.116 28100585 PMC5411768

[B62] Wang H. Li X. Wang Y. Tao Y. Lu S. Zhu X. (2018). Improvement of *n*-caproic acid production with *Ruminococcaceae* bacterium CPB6: selection of electron acceptors and carbon sources and optimization of the culture medium. *Microb. Cell Fact.* 17:99. 10.1186/s12934-018-0946-3 29940966 PMC6019802

[B63] Wick R. R. Judd L. M. Gorrie C. L. Holt K. E. (2017). Unicycler: resolving bacterial genome assemblies from short and long sequencing reads. *PLoS Comput. Biol.* 13:e1005595. 10.1371/journal.pcbi.1005595 28594827 PMC5481147

[B64] Xu J. Guzman J. J. L. Andersen S. J. Rabaey K. Angenent L. T. (2015). In-line and selective phase separation of medium-chain carboxylic acids using membrane electrolysis. *Chem. Commun.* 51 6847–6850. 10.1039/c5cc01897h 25792085

[B65] Xu J. Hao J. Guzman J. J. L. Spirito C. M. Harroff L. A. Angenent L. T. (2018). Temperature-phased conversion of acid whey waste into medium-chain carboxylic acids *via* lactic acid: no external e-donor. *Joule* 2 280–295. 10.1016/j.joule.2017.11.008

[B66] Yarza P. Yilmaz P. Pruesse E. Glöckner F. O. Ludwig W. Schleifer K.-H. (2014). Uniting the classification of cultured and uncultured bacteria and archaea using 16S rRNA gene sequences. *Nat. Rev. Microbiol.* 12 635–645. 10.1038/nrmicro3330 25118885

[B67] Zhu X. Zhou Y. Wang Y. Wu T. Li X. Li D. (2017). Production of high-concentration *n*-caproic acid from lactate through fermentation using a newly isolated *Ruminococcaceae* bacterium CPB6. *Biotechnol. Biofuels* 10:102. 10.1186/s13068-017-0788-y 28439295 PMC5399333

