# Supplementary material for: The Isolate Caproiciproducens sp. 7D4C2 Produces n-Caproate at Mildly Acidic Conditions From Hexoses: Genome and rBOX Comparison With Related Strains and Chain-Elongating Bacteria
Source: Front Microbiol. 2021 Jan 14;11:594524. doi: 10.3389/fmicb.2020.594524 (PMC7873966; doi:10.3389/fmicb.2020.594524)
Supplement: Supplementary Data Sheet 1 — Location and percent identity of all rBOX genes in strain 7D4C2, closely related bacteria, bacteria with similar rBOX genes, and known chain-elongating bacteria. In green: rBOX genes used in the phylogenetic analyses. [file Data_Sheet_1.PDF]

Supplementary Material

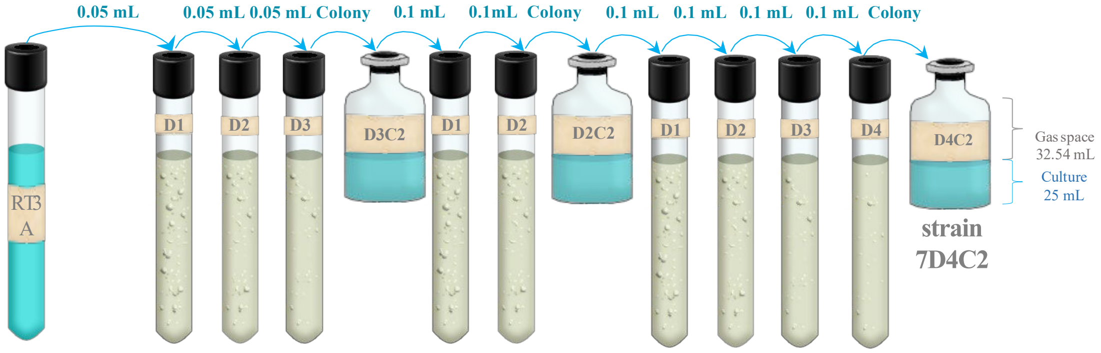

	Soft agar serial dilutions	Picked colony	Soft agar serial dilutions	Picked colony	Soft agar serial dilutions	Isolate
Ethanol, mM	50	50	-	-	-	-
Acetate, mM	-	-	-	-	5	5
n-Butyrate, mM	57.1	57.1	15	15	30	30
n-Caproate, mM	-	-	-	-	5	5
n-Caprylate, mM	1	1	-	-	-	-
Fructose, mM	55.5	55.5	30	30	30	30
Yeast extract, % w/v	0.05	0.05	0.05	0.05	0.3	0.3
Vitamin sol. 2x, % v/v	0.5	0.5	0.5	0.5	0.2	0.2
MES, mM	91.5	91.5	91.5	91.5	91.5	91.5
pH	5.2	5.2	5.2	5.2	5.2	5.2

Figure S1. Summary of the isolation process of strain 7D4C2. For the isolation, a sample from a chain-elongating reactor (SR1) with in-line product extraction - operated at a pH of 5.5 and a temperature of 30 ºC (Spirito, Angenent et al. unpublished work) - was enriched with ethanol (40 mM), acetate (4 mM), *n*-caproate (4 mM), and *n*-caprylate (4 mM) during 3 transfers (RT3A in the figure). This enriched culture was serially diluted in soft agar with supplemented basal medium (**Table S1**), as indicated in the figure. From the series of dilutions, an *n*-caproate producing colony was further diluted until its purity was confirmed *via* microscopy and Sanger Sequencing. RT3A: Reactor Transfer 3 Replicate A. D: dilution number. C: colony.

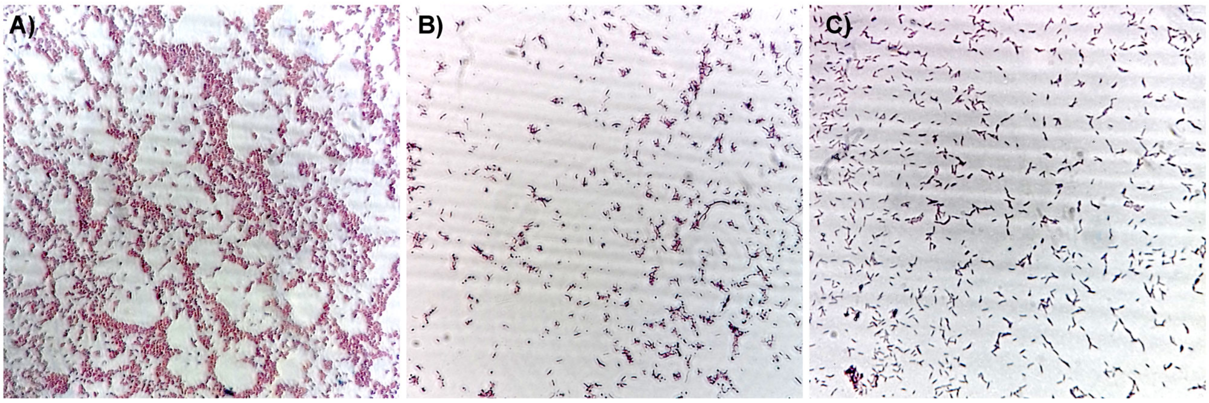

Figure S2. Gram staining of strain 7D4C2 and controls for negative and positive staining: **A)** *Escherichia colii* NEB® Stable, which is Gram negative); **B)** *Caproiciproducens galactitolivorans* BS-1, which is Gram positive; and **C)** strain 7D4C2, which is found here to be Gram positive.

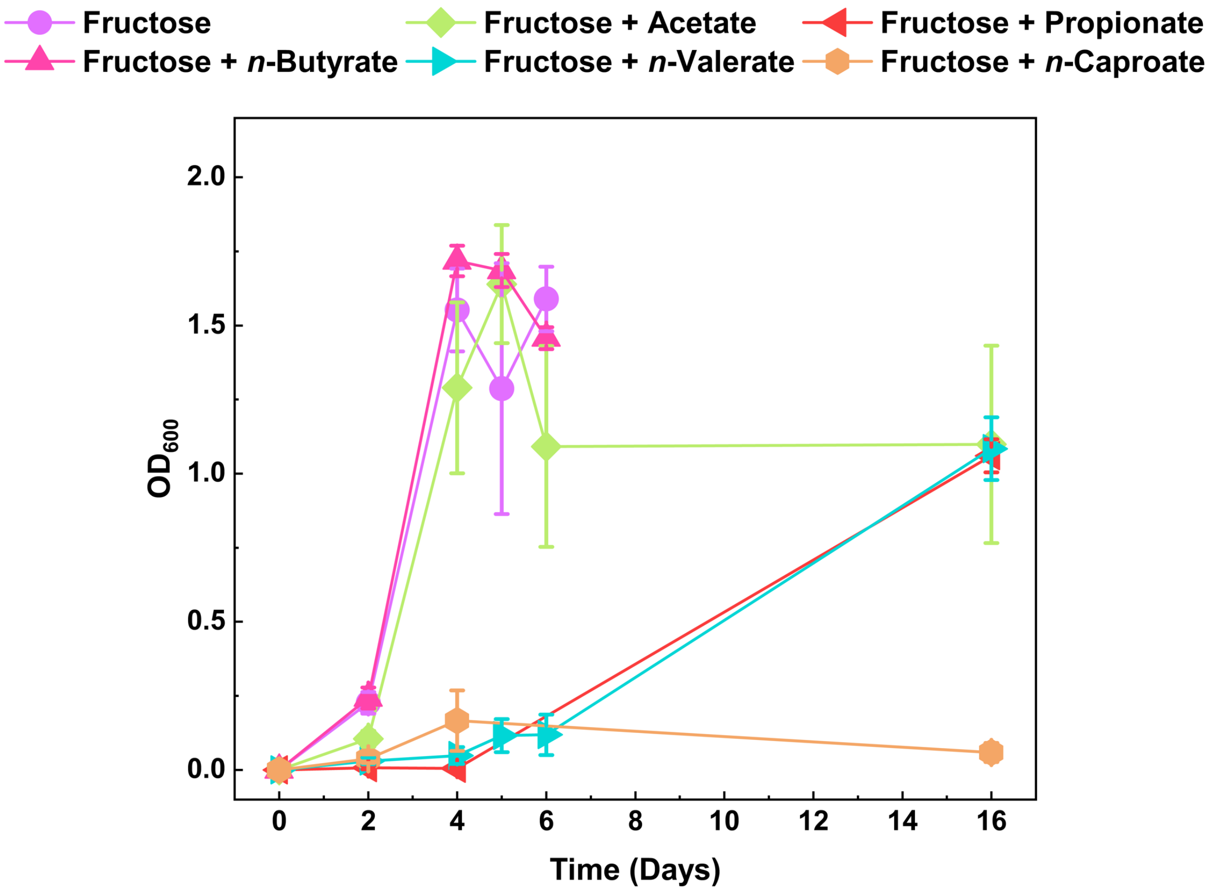

Figure S3 OD_{600} values measured throughout the culturing period for strain 7D4C2 with different electron acceptors (acetate, *n*-butyrate, propionate, *n*-valerate, and *n*-caproate) and fructose.

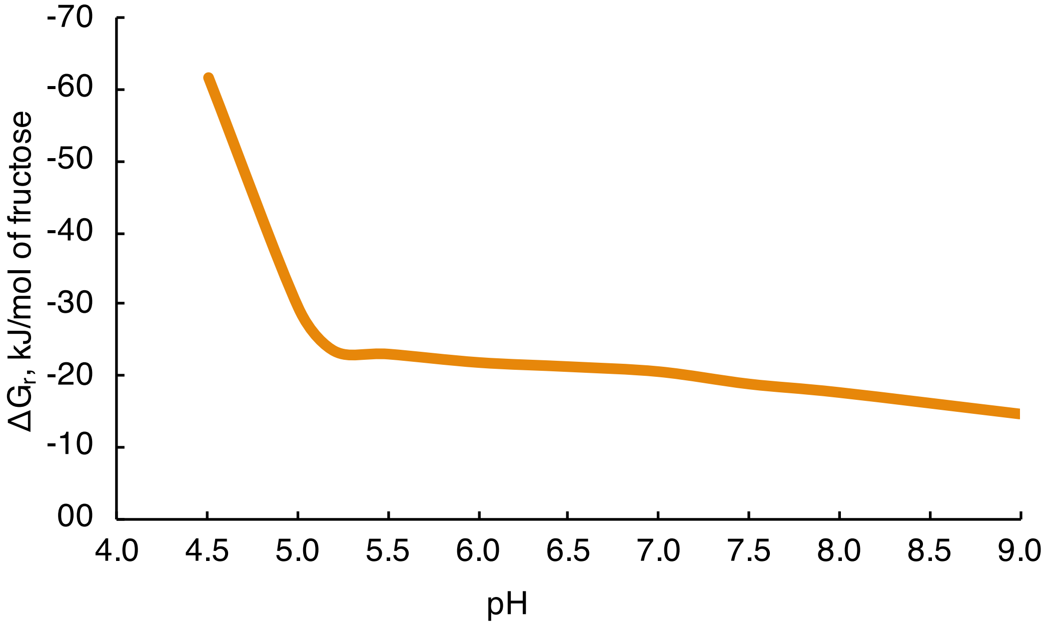

Figure S4 Actual Gibbs energy change (ΔG_r) for *n*-caproate production by strain 7D4C2 as a function of pH values. The stoichiometric equation estimated for caproate production from fructose and *n*-butyrate is $C_6H_{12}O_6 + C_4H_7O_2 + 2\ H_2O = C_6H_{11}O_2 + 4\ CO_2 + 12\ H^+ + 12\ e^-$. The concentration of products and fructose consumed and the H_2 partial pressures used for the calculations are summarized in **Table S3**. A partial pressure of 0.2 atm was assumed for CO_2. The equations used to calculate the ΔG_r values were those described in Rittmann and McCarty, 2001 (Chapter 2.6. Energetics and bacterial growth).

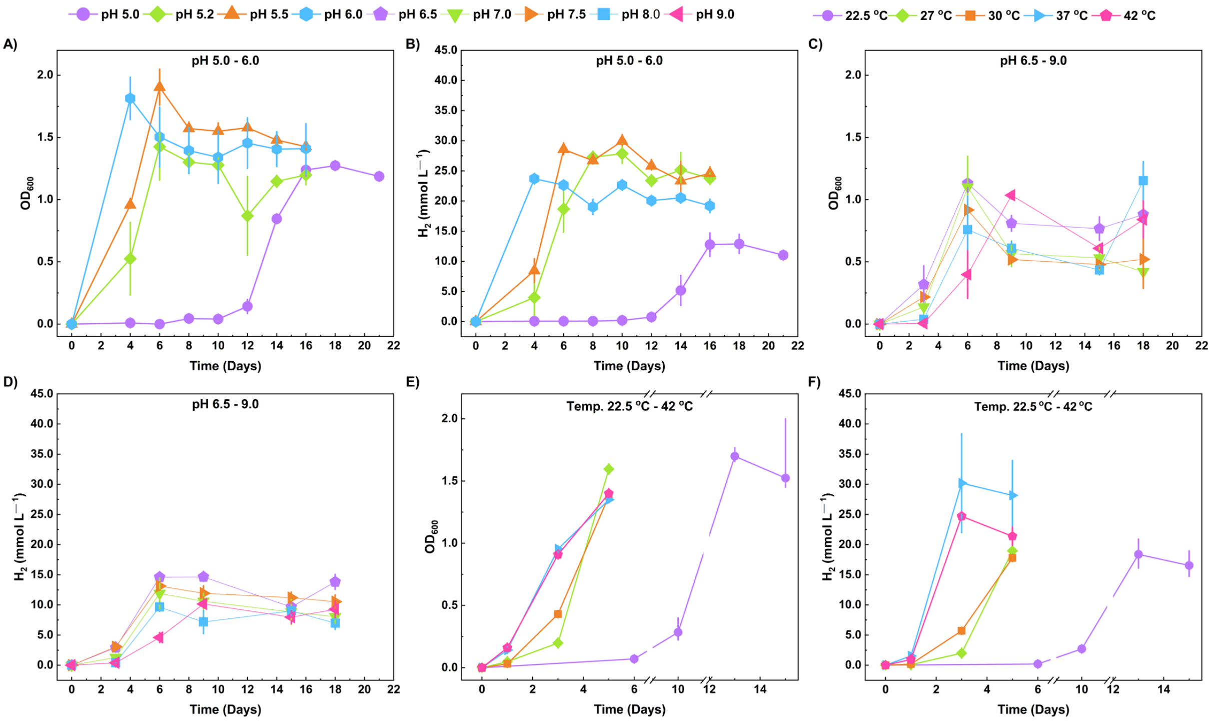

Figure S5. OD_{600} and H_2 production throughout the culturing period for strain 7D4C2 at different pH values and temperatures: **A)** OD_{600} at pH values of 5.0-6.0; **B)** H_2 production at pH values of 5.0-6.0; **C)** OD_{600} at pH values of 6.5 to 9.0; **D)** H_2 production at pH values of 6.5 to 9.0; **E)** OD_{600} at temperatures of 27 to 42 ºC; and **F)** H_2 production at temperatures of 27 to 42ºC. The OD_{600} and H_2 production of the test at a pH of 4.5 is not shown due to the extended lag phase of about 20 days. Bars represent minimum and maximum values between duplicate cultures.

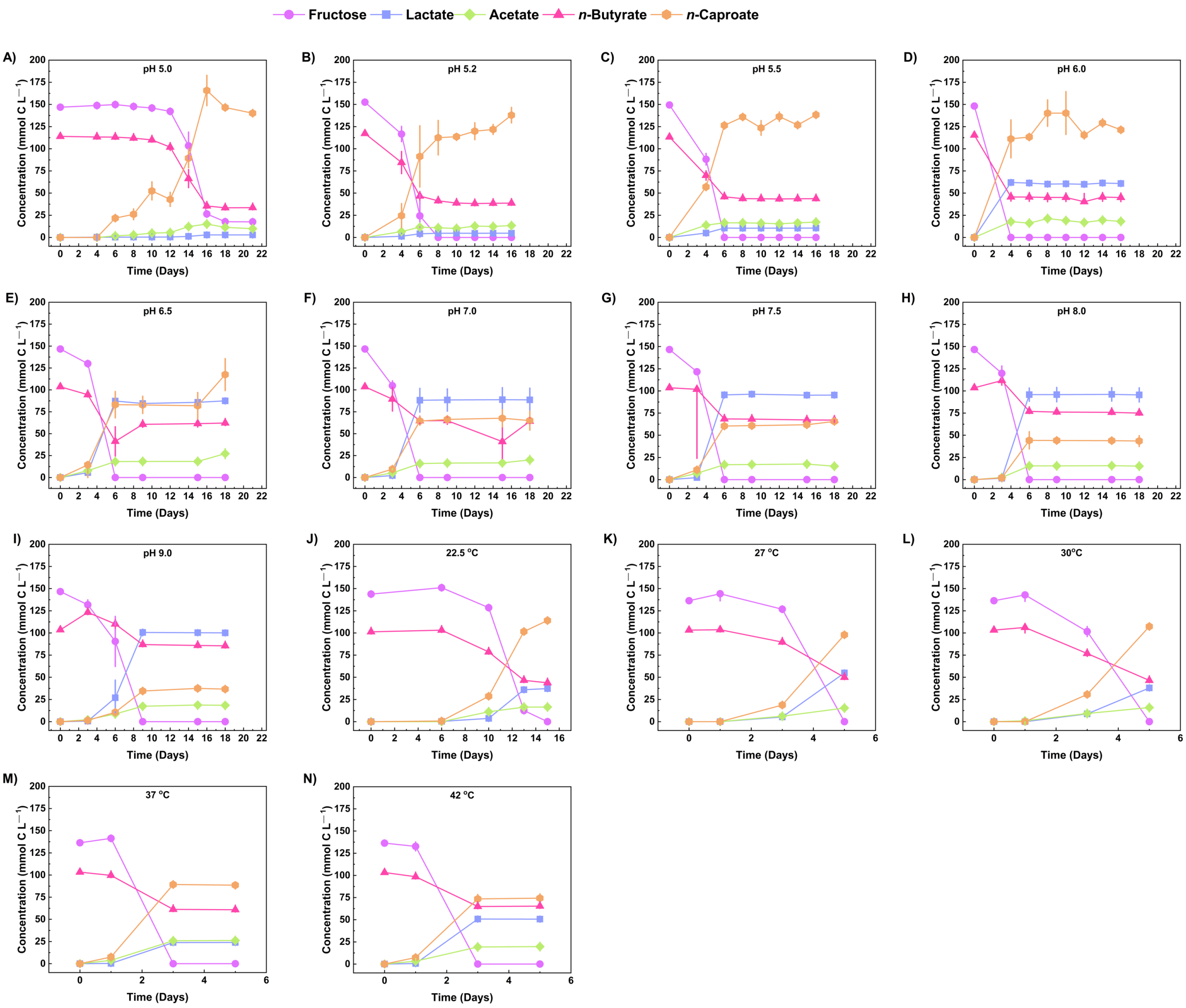

Figure S6. Fructose, *n*-butyrate, and products concentrations throughout the culturing period for strain 7D4C2 at different pH values and temperatures**: A-I)** graphs at different pH values. The graph at pH of 4.5 is not shown due to the extended lag phase of ~20 days; and **J-N)** graphs at different temperatures. Bars represent minimum and maximum values between duplicate cultures.

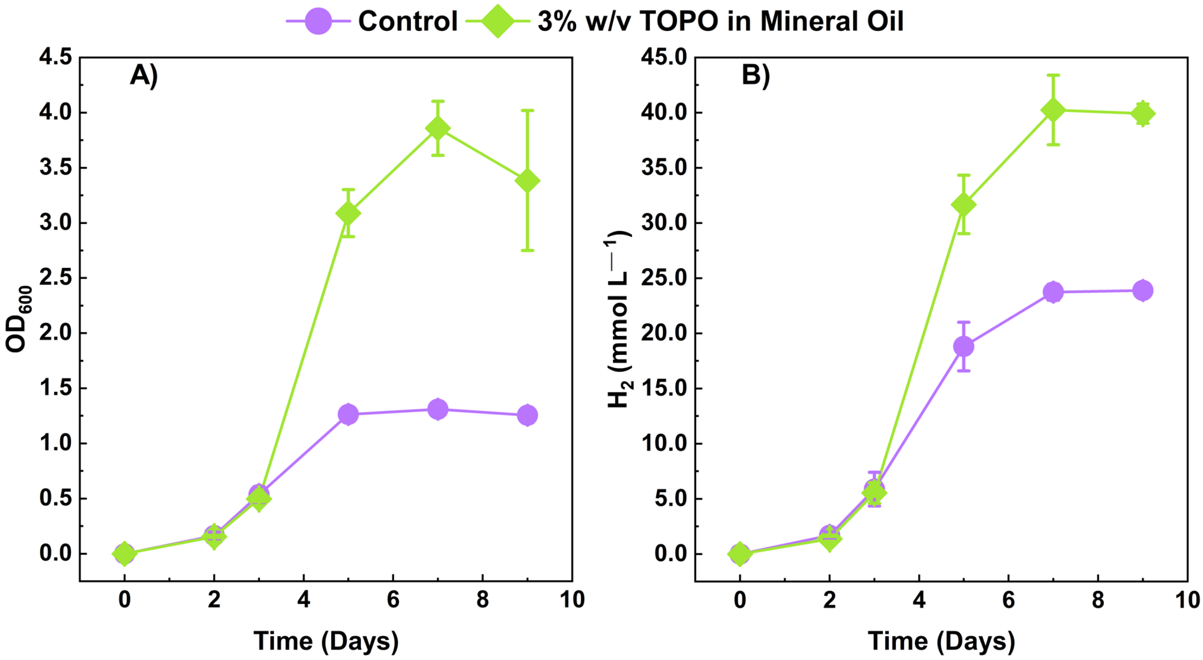

Figure S7. OD_{600} and H_2 production throughout the culturing period for strain 7D4C2 with and without extraction solvent**: A)** OD_{600}; and B) H_2 production. Error bars represent one standard deviation among triplicate cultures.

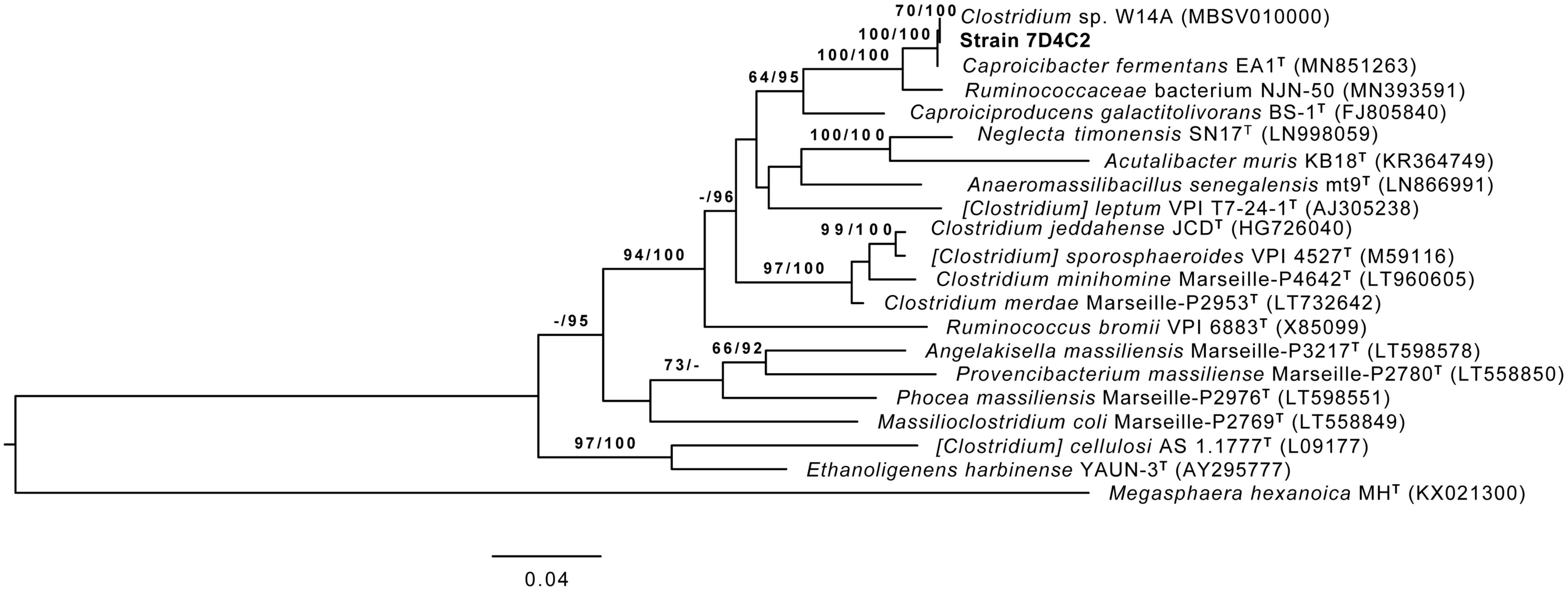

Figure S8. Phylogeny between strain 7D4C2 and its closest relatives based on the 16S rRNA gene. The branches are scaled in terms of the expected number of substitutions per site. The numbers above the branches are support values when larger than 60% from ML (left) and MP (right) bootstrapping. *Megasphaera hexanoica* MH^{T} (KX021300) was used as the outgroup. Bar indicates 4.0% sequence divergence. GenBank accession numbers of the 16S rRNA gene sequences are given in parentheses. The tree was built using one of the complete 16S rRNA genes found in the genome (NCBI PRJNA615378, 99.46% similar to the assembly done by Sanger Sequencing, NCBI MT056029).

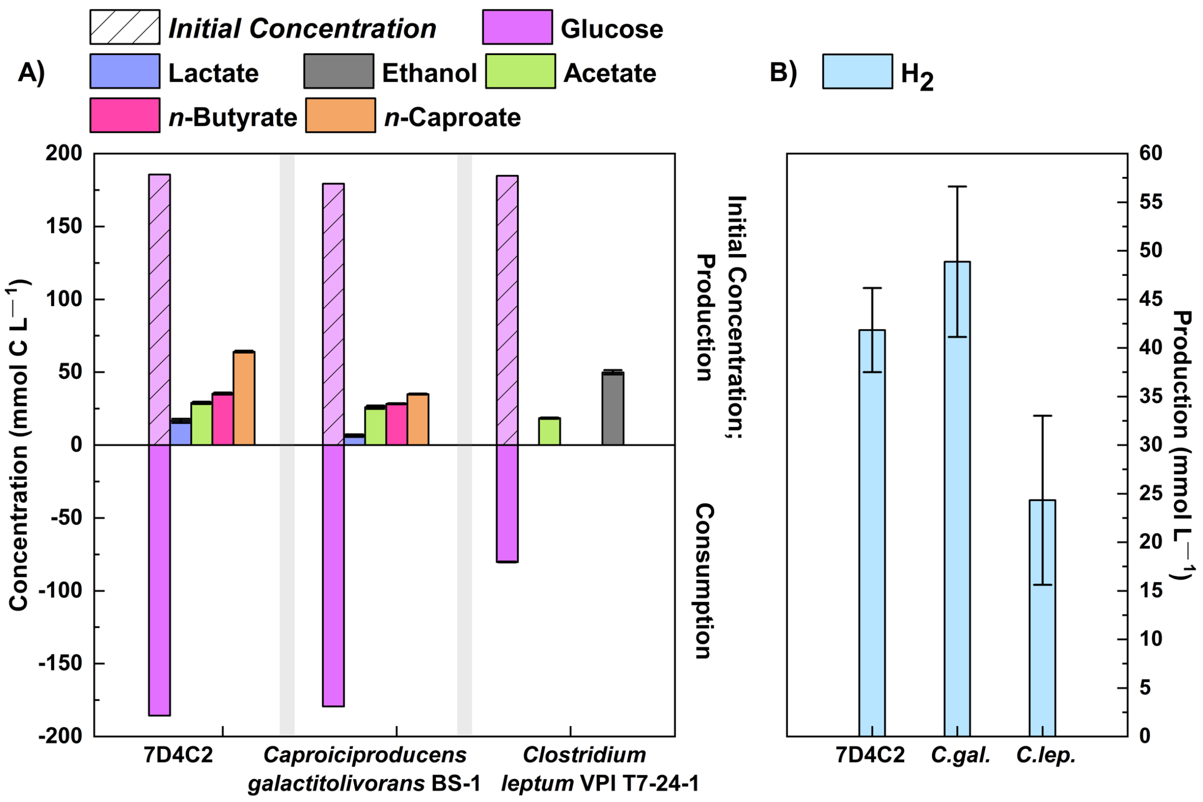

Figure S9. Comparison of glucose fermentation by strain 7D4C2, *C. galactitolivorans* and *C. leptum* in DSMZ medium 107c (pH 7.0 and 37ºC)**: A)** comparison of final products concentrations and glucose consumption between strains; and B) comparison of final H_2 production between strains. Error bars represent one standard deviation among triplicate cultures.

A)

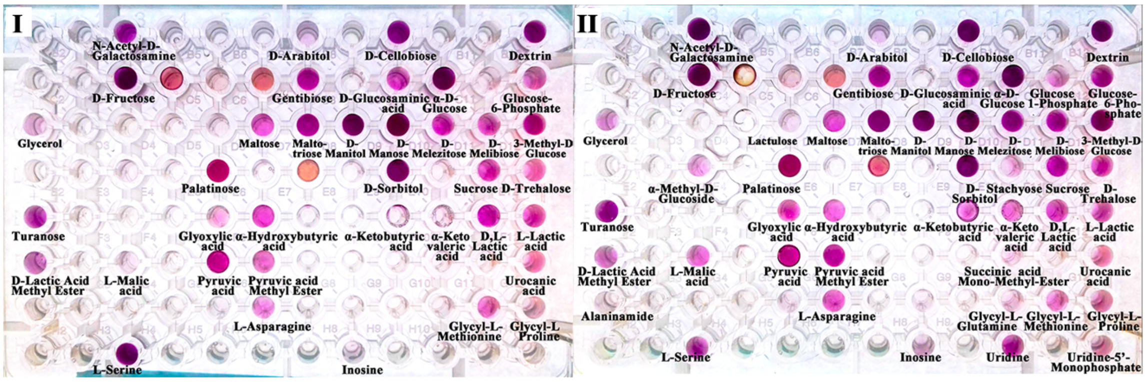

B)

Substrate	Test		Consumption	Substrate	Test		Consumption	Substrate	Test		Consumption
	I	II			I	II			I	II	
Water	0	0	N	D-Mannose	1	1	Y	Pyruvic Acid	1	1	Y
N-Acetyl-D- Galactosamine	0	0	N	D-Melezitose	1	1	Y	Pyruvic Acid Methyl Ester	1	1	Y
N-Acetyl-D- Glucosamine	1	1	Y	D-Melibiose	1	1	Y	D-Saccharic Acid	0	0	N
N-Acetyl-β-D- Mannosamine	0	0	N	3-Methyl-D- Glucose	1	1	Y	Succinamic Acid	0	0	N
Adonitol	0	0	N	α-Methyl-D- Galactoside	0	0	N	Succinic Acid	0	0	N
Amygdalin	0	0	N	β-Methyl-D- Galactoside	0	0	N	Succinic Acid Mono-Methyl Ester	0	1	N
D-Arabitol	1	1	Y	α-Methyl-D- Glucoside	0	1	N	m-Tartaric Acid	0	0	N
Arbutin	0	0	N	β-Methyl-D- Glucoside	0	0	N	Urocanic Acid	1	1	Y
D-Cellobiose	1	1	Y	Palatinose	1	1	Y	Alaninamide	0	1	N
α-Cyclodextrin	0	0	N	D-Raffinose	0	0	N	L-Alanine	0	0	N
β-Cyclodextrin	0	0	N	L-Rhamnose	0	0	N	L-Alanyl-L- Glutamine	0	0	N
Dextrin	1	1	Y	Salicin	0	0	N	L-Alanyl-L- Histidine	0	0	N
Dulcitol	0	0	N	D-Sorbitol	1	1	Y	L-Alanyl-L- Threonine	0	0	N
i-Erythritol	0	0	N	Stachyose	0	1	N	L-Asparagine	1	1	Y
D-Fructose	1	1	Y	Sucrose	1	1	Y	L-Glutamic Acid	0	0	N
L-Fucose	0	0	N	D-Trehalose	1	1	Y	L-Glutamine	0	0	N
D-Galactose	0	0	N	Turanose	1	1	Y	Glycyl-L- Aspartic Acid	0	0	N
D-Galacturonic Acid	0	0	N	Acetic Acid	0	0	N	Glycyl-L- Glutamine	0	1	N
Gentiobiose	1	1	Y	Formic Acid	0	0	N	Glycyl-L- Methionine	1	1	Y
D-Gluconic Acid	0	0	N	Fumaric Acid	0	0	N	Glycyl-L- Proline	1	1	Y
D-Glucosaminic Acid	1	1	Y	Glyoxylic Acid	1	1	Y	L-Methionine	0	0	N
α-D-Glucose	1	1	Y	α-Hydroxybutyric Acid	1	1	Y	L-Phenylalanine	0	0	N
α-D-Glucose-1-Phosphate	0	1	N	β- Hydroxybutyric Acid	0	0	N	L-Serine	1	1	Y
D-Glucose-6-Phosphate	1	1	Y	Itaconic Acid	0	0	N	L-Threonine	0	0	N
Glycerol	1	1	Y	α-Ketobutyric Acid	1	1	Y	L-Valine	0	0	N
D,L-α-Glycerol Phosphate	0	0	N	α-Ketovaleric Acid	1	1	Y	L-Valine plus L-Aspartic Acid	0	0	N
m-Inositol	0	0	N	D,L-Lactic Acid	1	1	Y	2'-Deoxy Adenosine	0	0	N
α-D-Lactose	0	0	N	L-Lactic Acid	1	1	Y	Inosine	1	1	Y
Lactulose	0	1	N	D-Lactic Acid Methyl Ester	1	1	Y	Thymidine	0	0	N
Maltose	1	1	Y	D-Malic Acid	0	0	N	Uridine	0	1	N
Maltotriose	1	1	Y	L-Malic Acid	1	1	Y	Thymidine-5'-Mono-phosphate	0	0	N
D-Mannitol	1	1	Y	Propionic Acid	0	0	N	Uridine-5'- Mono-phosphate	0	1	N

Figure S10. Substrate consumption by strain 7D4C2 according to the AN MicroPlate™ from Biolog (Hayward, CA)**: A)** pictures of the duplicate assays (I and II), where purple wells indicate oxidation of the substrate; and **B)** summary of results. 1 and 0 indicate positive and negative oxidation, respectively, in each test (I and II). Only when both tests showed positive oxidation, the substrate was considered to be metabolized (Y).

frontiers

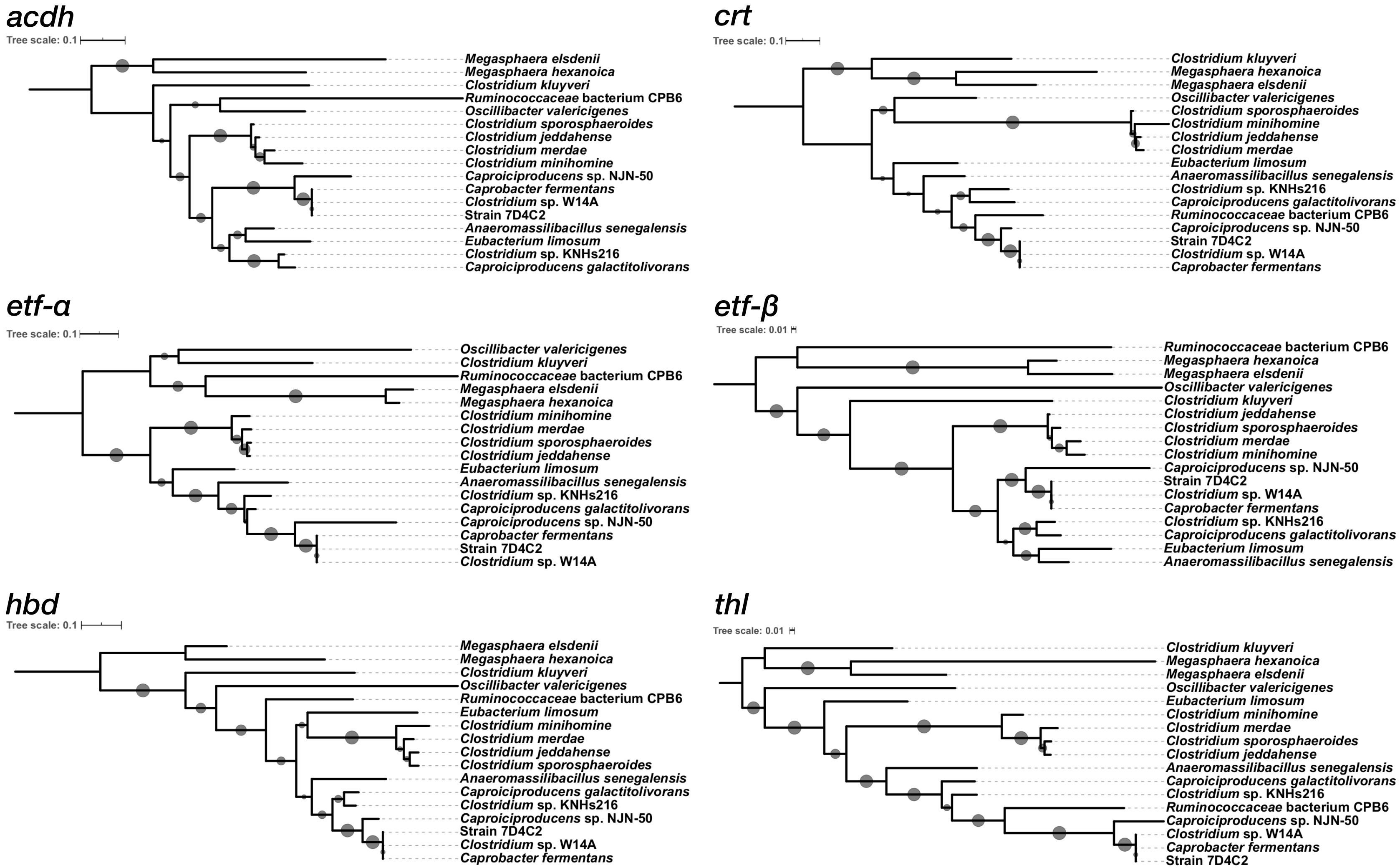

Figure S11. Phylogenetic trees of the rBOX genes in strain 7D4C2, closest relatives and known chain-elongating bacteria. The genes of *Clostridium kluyveri* are used as outgroups. The multiple sequence alignments were computed by MUSCLE and the gene trees were inferred by RAxML with 1000 iterations of bootstrapping (values greater than 30% are shown as circles on the edges) and visualized by iTOL.

Table S1 Composition of the supplemented basal medium, the modified Wolfe's trace minerals solution, and the vitamin solution. For the serial dilutions in soft agar, the medium contained the substrates as summarized in **Figure S1**.

**1. Supplemented Basal medium (1 L)**		**2. Modified Wolfe's Trace minerals (1 L)**		**3. Vitamin solution 2x (1 L)**	
NaCl	1.1 g	EDTA	500 mg	Pyridoxine hydrochloride	20 mg
NH_4Cl	0.474 g	$MgCl_2 \cdot 6H_2O$	3.63 g	Riboflavin	10 mg
$CaCl_2 \cdot 2H_2O$	0.1 g	$MnCl_2 \cdot 4H_2O$	427.1 mg	Nicotinic acid	10 mg
$MgCl_2 \cdot 6H_2O$	0.065 g	NaCl	1 g	Folic acid	4 mg
KH_2PO_4	0.15 g	$CoCl_2 \cdot 6H_2O$	180 mg	Calcium pantothenate	10 mg
Na_2CO_3	0.032 g	$CaCl_2$ (anhydrous)	100 mg	P-amino benzoic acid	10 mg
Trace mineral Wolfe modified	20 mL	$ZnCl_2$	210.9 mg	D-biotin	4 mg
Na-Butyrate	3.3 g	$CuCl_2 \cdot 2H_2O$	14.64 mg	Thioctic acid	10 mg
DI H_2O	856 mL	H_3BO_3	10 mg	Vitamin B12	10 mg
**Sparge 30-40 min N_2:CO_2 (80:20)**		$Na_2MoO_4 \cdot 2H_2O$	10 mg	Thiamine HCl	10 mg
L-cysteine HCl	0.47 g	Na_2SeO_3 (anhydrous)	1 mg	2-mercaptoethanesulfonic acid	4 mg
$FeCl_2$ (10g/L stock solution)	0.125 mL	$Na_2WO_4 \cdot 2H_2O$	10 mg		
**Autoclave 121°C, 20min**		$NiCl_2 \cdot 6H_2O$	20 mg		
Yeast extract (10% w/v)[a]	20 mL				
Fructose or glucose (250 g/L)[a]	22 mL				
Vitamin solution 2x[a]	2 mL				
MES 1M[a]	100 mL				
**Adjust to pH 5.5-5.6**					

[a]filter sterilized

Table S2. Maximum OD_{600}, final electron donor and acceptor and product concentrations, and *n*-caproate specificity in cultures of strain 7D4C2 with different electron acceptors and for the control with only fructose.

Electron donor and acceptor	Max OD_{600}	H_2 production (mmol L^{-1})	Lactate concentration (mmol C L^{-1})	Acetate concentration (mmol C L^{-1})	Propionate concentration (mmol C L^{-1})	*n*-Butyrate concentration (mmol C L^{-1})	*n*-Valerate concentration (mmol C L^{-1})	*n*-Caproate concentration (mmol C L^{-1})	*n*-Caproate carboxylate specificity (%)
Fruc	1.59 ± 0.11	22.49 ± 2.06	25.42 ± 2.69	4.56 ± 0.64	ND	6.93 ± 0.59	ND	57.52 ± 2.37	60.90 ± 1.51
Fruc + C2	1.64 ± 0.20	14.33 ± 0.42	10.20 ± 2.41	-14.26 ± 8.33	ND	38.66 ± 7.15	ND	40.27 ± 15.41	44.08 ± 5.90
Fruc + C3	1.06 ± 0.06	NM	5.01 ± 2.76	15.60 ± 0.52	-57.53 ± 2.03	6.67 ± 0.59	76.51 ± 0.40	2.99 ± 0.64	2.79 ± 0.52
Fruc + C4	1.69 ± 0.06	27.56 ± 2.80	6.25 ± 1.05	19.31 ± 0.54	ND	-55.13 ± 1.15	ND	125.49 ±1.89	83.08 ± 0.44
Fruc + C5	1.08 ± 0.11	NM	46.15 ± 3.15	8.78 ± 1.15	ND	7.04 ± 1.05	-10.95 ± 0.78	44.31 ± 5.3	41.43 ± 3.33
Fruc + C6	0.17 ± 0.1	NM	4.02 ± 1.26	ND	ND	ND	ND	-1.96 ± 0.54	NP

Positive values represent production and negative values represent consumption. Error represents one standard deviation among triplicates. ND: not detected; NM: not measured; NP: not produced. The pH value of the test was 5.5 ± 0.02. Fruc: fructose; C2: acetate; C3: propionate; C4: *n*-butyrate; C5: *n*-valerate; C6: *n*-caproate.

Table S3. Maximum OD_{600} values, final concentration of products, and specificities of lactate and *n*-caproate in cultures of strain 7D4C2 grown at different pH values and temperatures.

pH / Temp (/ ^{0}C)	Max OD_{600}	Growth rate* (d^{-1})	PH_2 (atm)	H_2 production (mmol L^{-1})	Lactate concentration (mmol C L^{-1})	Acetate concentration (mmol C L^{-1})	*n*-Butyrate concentration (mmol C L^{-1})	*n*-Caproate concentration (mmol C L^{-1})	Lactate carboxylate specificity (%)	*n*-Caproate carboxylate specificity (%)	Average fructose consumption (mmol C L^{-1} d^{-1})
4.5 / 30	0.43	NA	0.00	4.40	0.00 0.00	8.15 10.6	-57.02 -51.18	93.18 108.24	0.00 0.00	91.08 92.78	-1.9 -0.2
5.0 / 30	1.25 1.29	0.63	0.05	10.74 11.29	2.01 3.753	9.66 10.09	-81.96 -78.6	138.02 142.36	1.30 2.47	90.88 92.42	-7.32 -7.03
5.2 / 30	1.15 1.69	0.50	0.12	23.22 24.29	4.58 5.00	12.49 14.33	-78.55 -78.41	129.16 146.66	3.01 3.13	88.32 88.36	-17.34 -25.39
5.5 / 30	1.56 1.58	0.34	0.12	24.28 24.89	9.23 11.91	17.14 17.56	-70.24 -68.86	137.04 139.69	5.55 6.36	82.51 83.91	-24.91 -24.91
6.0 / 30	1.64 1.98	1.3	0.09	18.09 20.32	60.52 61.16	17.94 18.38	-75.03 -65.75	119.81 123.03	30.26 30.45	60.30 60.87	-37.04 -37.04
6.5 / 30	1.03 1.23	0.42	0.07	12.58 15.07	84.62 90.39	21.44 32.90	-44.41 -38.44	99.24 135.72	33.41 42.82	47.02 53.59	-24.45 -24.45
7.0 / 30	0.85 1.35	0.69	0.04	7.98 8.11	75.05 102.04	14.81 25.29	-43.37 -35.43	54.02 75.88	50.22 52.16	37.34 37.54	-24.45 -24.45
7.5 / 30	0.90 0.93	0.48	0.05	9.41 11.67	93.50 97.03	11.17 18.90	-36.55 -36.37	62.83 68.45	52.62 55.82	37.12 37.51	-24.45 -24.45
8.0 / 30	1.00 1.31	0.98	0.04	5.94 8.01	87.51 103.54	13.59 16.69	-29.86 -27.34	37.28 49.67	60.94 63.24	26.94 29.23	-24.45 -24.45
9.0 / 30	1.02 1.05	0.85	0.04	7.94 10.63	97.12 103.01	17.34 19.60	-19.48 -16.70	35.64 37.77	64.23 64.70	23.55 23.75	-16.30 -16.30
6.0 / 22.5	1.66 1.77	0.45	0.08	14.72 18.97	35.84 39.52	16.07 17.08	-60.12 -54.62	113.57 115.01	21.61 23.17	67.41 68.57	-10.14 -9.78
6.0 / 27	1.59 1.61	0.88	0.09	18.42 19.52	52.43 57.52	15.25 15.55	-54.14 -52.54	96.32 99.62	31.34 33.95	56.86 59.55	-27.29 -27.29
6.0 / 30	1.37 1.39	0.95	0.09	17.66 17.94	38.03 38.07	15.87 15.95	-56.75 -56.72	105.83 108.94	23.36 23.81	66.26 66.85	-27.29 -27.29
6.0 / 37	1.35 1.36	0.96	0.14	22.41 33.94	21.44 26.42	26.13 26.25	-43.84 -40.97	88.53 88.54	15.75 18.71	62.70 65.05	-45.48 0.45.48
6.0 / 42	1.40 1.40	0.87	0.11	19.87 22.90	50.39 50.93	19.20 20.11	-39.41 -36.38	69.44 79.18	33.90 36.24	49.95 52.71	-45.48 0.45.48

Negative values denote consumption. PH_2: H_2 partial pressure (1 atm = 101.325 kPa). NA: not available
*Growth rate given for the exponential phase (**see Figure S5**), calculated by plotting Ln(OD_{600}) *vs*. time.

Table S4. Average nucleotide identity (ANI) and alignment fraction (AF) values between strain 7D4C2 and the most similar type strains of the order Clostridiales (heterotypic synonym of Eubacteriales) and unclassified species which are closely related; as calculated by JSpeciesWS.

**Genome**	**ANI**	**AF**
Clostridium sp. W14A	97.64	82.28
Caproicibacter fermentans EA1	97.34	81.20
Caproiciproducens sp. NJN-50	78.52	44.36
Clostridium sp. KNHs216	71.14	32.50
Ethanoligenens harbinense YUAN-3	69.65	15.30
Caproiciproducens galactitolivorans BS-1	69.34	28.22
Anaeromassilibacillus senegalensis mt9	69.21	21.65
Oscillibacter valericigenes Sjm18-20	68.74	16.15
Clostridium jeddahense JCD	68.30	22.78
[Clostridium sporosphaeroides VPI 4527	68.20	23.71
[Clostridium] leptum VPI T7-24-1	67.55	15.78
Clostridium minihomine Marseille-P4642	67.40	19.62
Clostridium merdae Marseille-P2953	66.78	19.09
Clostridium kluyveri K1	65.32	6.05
Christensenella minuta DSM 22607	64.67	8.00
Eubacterium limosum SA11	64.11	8.54

Table S5. Comparison of carbohydrates oxidized by strain 7D4C2, *C. galactitolivorans*, and *C. fermentans*.

**Substrate**	**strain 7D4C2**	***C. galactitolivorans***	***C. fermentans***
N-Acetyl-D- Glucosamine	+	-	
Adonitol	-	-	
Amygdalin	-	-	
D-Arabitol	+	-	
Arbutin	-	-	
D-Cellobiose	+	+	+
Dextrin	+	+	
Dulcitol	-	+	
i-Erythritol	-	-	
D-Fructose	+	+	+
L-Fucose	-	+	
D-Galactose	-	+	+
Gentiobiose	+	-	
D-Gluconic Acid	-	-	
α-D-Glucose	+	+	
Glycerol	+	+	-
m-Inositol	-	+	
α-D-Lactose	-	-	
Maltose	+	-	
D-Mannitol	+	+	+
D-Mannose	+	+	+
D-Melezitose	+	-	
D-Melibiose	+	-	
D-Raffinose	-	-	
L-Rhamnose	-	-	
Salicin	-	-	
D-Sorbitol	+	+	+
Sucrose	+	-	
D-Trehalose	+	-	
Turanose	+	-	

"+" (highlighted in green) indicate that a substrate is metabolized and **"-"** indicate that a substrate is not metabolized, according to the AN MicroPlate^{TM} from Biolog (Hayward, CA) for strain 7D4C2 (**Figure S10**), and the data reported for *C. galactitolivorans* in Kim *et al.*, 2015 and for *C. fermentans* in Flaiz *et al*. Blank: not reported.
